# A systems herbal-to-molecule and transcriptomic strategy identifies emodin as an ESR1-targeting phytochemical driving thrombopoiesis

**DOI:** 10.1186/s13020-026-01454-5

**Published:** 2026-07-06

**Authors:** Xiao Qi, Qinyao Li, Fengyu Li, Linglin Zhou, Qi Mo, Jing Zeng, Tianci Hu, Sheng Liu, Xinyue Mei, Min Wu, Xuejing Qiang, Qiyang Cheng, Anguo Wu, Xiaogang Zhou, Feihong Huang, Qiaozhi Wang, Peng Chen, Jianming Wu, Long Wang

**Affiliations:** 1 Department of Pharmacology, School of Pharmacy, Southwest Medical University, Luzhou, 646000 Sichuan China; 2 Department of Pharmacy, Chengdu Eighth People’s Hospital, Geriatric Hospital of Chengdu Medical College, Chengdu, 610083 Sichuan China; 3 Department of Pharmacy, Xichang People’s Hospital, Xichang, Liangshan Yi Autonomous Prefecture, Xichang, 615000 Sichuan China; 4 Basic Medical School, Guizhou University of Traditional Chinese Medicine, Guiyang, 550000 Guizhou China; 5 School of Basic Medical Sciences, Southwest Medical University, Luzhou, 646000 Sichuan China; 6 Education Ministry Key Laboratory of Medical Electrophysiology, Sichuan Key Medical Laboratory of New Drug Discovery and Druggability Evaluation, Luzhou Key Laboratory of Activity Screening and Druggability Evaluation for Chinese Materia Medica, Southwest Medical University, Luzhou, 646000 Sichuan China

**Keywords:** *Rhei Radix et Rhizoma*, Emodin, Megakaryocyte differentiation, Thrombopoiesis, ESR1

## Abstract

Radiation-induced thrombocytopenia (RIT) is a major dose-limiting toxicity of radiotherapy, for which effective pharmacological interventions remain limited. *Rhei Radix et Rhizoma* (RR) is widely used in traditional medicine, yet its thrombopoietic activity and molecular basis have not been systematically defined. Here, we investigated the effects of RR on thrombopoiesis using an integrated herbal-to-molecule and transcriptomic strategy. RR extract and representative phytochemicals were screened for megakaryocyte (MK) differentiation in Meg-01, K562, and murine haematopoietic stem and progenitor cells (HSPCs), followed by validation in a mouse RIT model. Network pharmacology and transcriptomic analyses indicated that RR regulates thrombopoiesis through coordinated multi-pathway modulation. Emodin was identified as the principal bioactive constituent mediating these effects. Functionally, emodin promoted MK maturation and polyploidization in vitro, accelerated megakaryopoiesis and platelet recovery in irradiated mice. Integrated target prediction and experimental validation identified ESR1 as a direct functional target of emodin, supported by molecular docking, molecular dynamics simulations, CETSA, and DARTS assays. Mechanistically, emodin activated ESR1-dependent PI3K/AKT and JAK2/STAT3 signaling, leading to induction of lineage-specific transcription factors and enhanced platelet biogenesis. Collectively, these findings identify emodin as a key thrombopoietic constituent of RR and reveal an ESR1-centered regulatory axis in megakaryopoiesis, supporting its therapeutic potential for RIT.

**Graphical Abstract:**

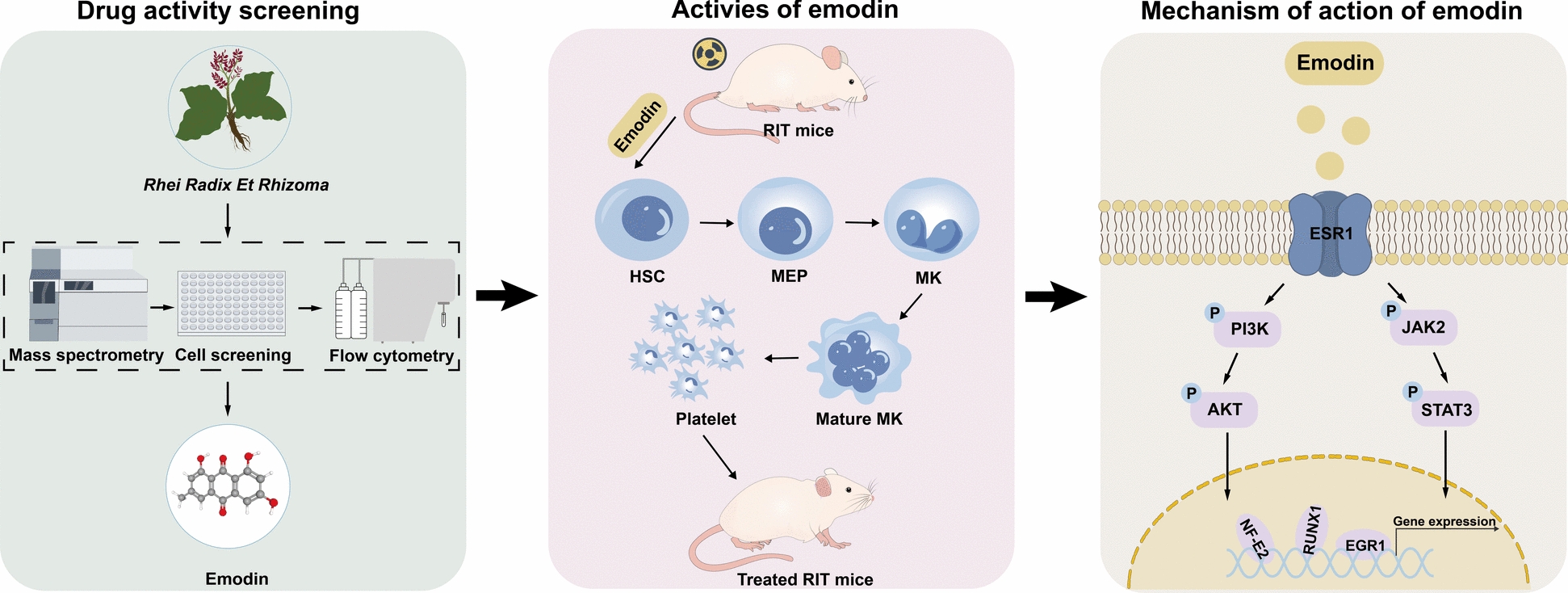

**Supplementary Information:**

The online version contains supplementary material available at 10.1186/s13020-026-01454-5.

## Introduction

Exposure to ionizing radiation remains an unavoidable hazard in several modern settings, including nuclear technology, aerospace missions, and clinical oncology, where radiotherapy continues to serve as a major therapeutic modality [1]. A primary consequence of moderate to high radiation exposure is profound myelosuppression, which leads to pancytopenia [2]. Among these hematological complications, thrombocytopenia is one of the most frequent and clinically serious, markedly increasing the risk of life-threatening hemorrhage and often forcing interruption of anticancer treatment [3]. Current therapeutic options, such as platelet transfusion and thrombopoietin (TPO) mimetics including recombinant human TPO (rhTPO) and eltrombopag, provide short-term benefit but remain limited by donor dependence, transient efficacy, and notable safety concerns such as bone marrow (BM) fibrosis and thrombotic events. Moreover, patients who are refractory or insensitive to TPO signaling remain difficult to treat [4–6]. These limitations underscore the need to develop novel thrombopoietic agents that operate through TPO-independent or mechanistically complementary pathways.

Megakaryopoiesis is a highly coordinated process in which hematopoietic progenitors undergo lineage commitment, endomitosis, cytoplasmic maturation, and ultimately release platelets into circulation [7]. TPO/c-MPL-JAK2/STAT3 axis is the primary pathway of megakaryocyte (MK) proliferation and polyploidization, while additional pathways-including PI3K/AKT and MEK/ERK-play essential roles in MK survival, cytoskeletal remodeling, and terminal platelet formation [8]. Identifying alternative regulatory nodes within this network has therefore become an important strategy for expanding therapeutic approaches to thrombocytopenia. Recent findings have renewed interest in estrogen-related signaling because of its possible involvement in hematopoietic regulation [9]. Although estrogen receptors are expressed in MKs and platelets, previous research has focused mainly on ERβ, and the physiological and pharmacological roles of ESR1 (ERα) in MK biology remain largely undefined [10]. Emerging evidence suggests that ESR1 may influence hematopoietic processes, yet its specific involvement in MK lineage commitment, polyploidization, and thrombopoiesis has not been systematically examined. Importantly, no thrombopoietic agent currently approved or in development has been designed to enhance platelet production through ESR1-centered mechanisms. Given the known interactions between ESR1 and key MK-related pathways such as PI3K/AKT and JAK2/STAT3 in other cellular systems, ESR1 represents an unexplored but potentially druggable regulatory node that could support the development of new TPO-independent strategies [11, 12].

Traditional Chinese Medicine (TCM) has been historically applied to alleviate bone marrow suppression and restore hematopoietic balance [13]. Among these classical remedies, *Caulis Polygoni Multiflori* and *Rhizoma Cibotii* have shown significant thrombopoietic activity in radiation-induced thrombocytopenia (RIT), accelerating MK differentiation and platelet recovery [14, 15]. Building on these findings, we extended our investigations to identify bioactive small molecules responsible for thrombopoietic effects. By applying an artificial intelligence-guided gcForest screening strategy, we identified 3, 8-di-O-methylellagic acid 2-O-glucoside as a enhancer of MK maturation through an ERK/HIF-1β/NF-E2-dependent, TPO-independent pathway [16]. In parallel, we discovered 3, 4, 3′-tri-O-methylellagic acid, a natural agonist of the TPO receptor that activates mTOR and ERK signaling to promote thrombopoiesis [17]. These studies highlight the ability of natural products to modulate both classical and non-classical thrombopoietic signaling pathways and emphasize their value as a source of chemical leads for drug development. Expanding on our efforts to integrate empirical herbal efficacy with defined molecular mechanisms, we next focused on *Rhei Radix et Rhizoma* (RR), a classical TCM herb historically used for detoxification and hematological disorders [18, 19]. Emodin, one of its predominant anthraquinone constituents, is recognized for its anti-inflammatory, antioxidant, analgesic, and cytoprotective activities [20–23]. However, despite its broad pharmacological actions, the hematopoietic effects of RR remain insufficiently characterized, and the specific contribution of emodin to MK development remains unclear.

Here, we employed a systematic, stepwise research strategy to delineate the thrombopoietic potential of RR and its active constituents. We first evaluated the efficacy of RR in RIT mice and demonstrated its ability to enhance MK differentiation in vitro. Guided by these findings, we conducted component profiling and functional screening and identified emodin as a major bioactive molecule responsible for the thrombopoietic activity of RR. Mechanistic investigations revealed that emodin markedly promotes MK differentiation, polyploidization, and maturation and significantly accelerates platelet recovery following radiation-induced BM injury. Most notably, Emodin exerts these effects through coordinated activation of ESR1-PI3K/AKT and JAK2/STAT3 signaling pathways, establishing ESR1 as a previously unrecognized regulatory hub in megakaryopoiesis. Together, this work not only provides molecular evidence supporting the traditional hematological use of RR but also demonstrates a comprehensive research paradigm that progresses from herbal extracts to active natural molecules and finally to target identification. By defining ESR1 as a novel, druggable node in thrombopoiesis, this study highlights emodin as a viable lead compound for development of next-generation, TPO-independent thrombopoietic agents.

## Materials and methods

### Chemicals

RR extract was obtained from Sichuan Neo-Green Pharmaceutical Technology Development Co., Ltd. Emodin (purity ≥ 98%) was purchased from Shanghai Acmec Biochemical Technology Co., Ltd.

### Chemical profiling of RR

Chemical constituents of RR were detected by ultra-high performance liquid chromatography–high-resolution mass spectrometry (UHPLC–HRMS) system. RR granules were dissolved in 70% methanol, sonicated for 30 min, and centrifuged at 12,000 × *g* for 10 min; the resulting supernatant was filtered through a 0.22-µm membrane prior to injection. Chromatographic separation was performed on an Exion UHPLC system (SCIEX, MA, USA) equipped with an Agilent SB-C18 column (100 mm × 2.1 mm, 2.7 μm). The mobile phase consisted of water containing 0.1% formic acid (solvent A) and acetonitrile (solvent B), delivered at 0.3 mL/min under the following gradient: 5% B from 0–1 min, linearly increased to 50% B from 1–18 min, then to 100% B from 18–20 min, and held at 100% B from 20–25 min. The column temperature was maintained at 40 °C. Mass spectrometric detection was performed on an X500R Q-TOF (SCIEX) operated in negative electrospray ionization (ESI) mode, with an ion source temperature of 500 °C, nebulizer gas (Gas 1) and heater gas (Gas 2) pressures at 50 psi, curtain gas at 35 psi, and CAD gas at 7 psi. Data were acquired in information-dependent acquisition (IDA) mode, including full-scan MS and MS/MS spectra obtained with collision-induced dissociation at 40 ± 15 V. Up to 10 precursor ions were selected per cycle using an intensity threshold of 400 cps across a mass range of 100–1500 Da, with an ion spray voltage of − 5500 V and a collision energy spread of 15 V. Detected ions were annotated based on accurate masses, retention times, and MS/MS fragmentation patterns, providing the chemical basis for subsequent network pharmacology analyses.

### Quantification of emodin in RR by LC–MS/MS

LC–MS/MS analyses were performed using an Agilent 1290 Infinity II ultra-high-performance liquid chromatography system coupled with an Agilent 6470B triple quadrupole mass spectrometer. Chromatographic separation was carried out on an InfinityLab Poroshell 120 SB-C_18_ column (100 × 2.1 mm, 2.7 μm) maintained at 40 °C. The mobile phase consisted of A (0.1% formic acid in water) and B (acetonitrile). The elution gradient was programmed as follows: 0–2 min, 30% B; 2–3 min, 30–50% B; 3–4 min, 50–70% B; 4–6 min, 70–90% B; 6 min, 90% B. The flow rate was set at 0.3 mL/min, and the injection volume was 5 μL. Mass spectrometric detection was performed in the negative electrospray ionization (ESI⁻) mode using multiple reaction monitoring (MRM). The MRM transitions for the target analyte were m/z 269 → 241.1 (collision energy: 35 V) and m/z 269 → 225.0 (collision energy: 25 V). Ion source parameters were optimized as follows: gas temperature, 400 °C; gas flow, 5 L/min; nebulizer pressure, 30 psi; sheath gas temperature, 350 °C; sheath gas flow, 12 L/min; and capillary voltage, 3 kV. For sample preparation, a total of 10.57 mg of RR were dissolved in 4 mL of water. An appropriate amount of the sample solution was filtered and subjected to instrumental analysis. For standard curve preparation, an emodin stock solution (0.5 mg/mL) was prepared in methanol. Working solutions were then diluted with water to concentrations of 50, 80, 100, 250, 400, and 2000 ng/mL for the establishment of a calibration curve. The calibration curve was used for quantitative determination of emodin in RR.

### Cell culture

K562 and Meg-01 cells were cultured in complete RPMI-1640 medium at 37 °C.

### Induction of mouse BM haematopoietic stem and progenitor cells into MKs

BM cells passed through a 70-μm strainer, and haematopoietic stem and progenitor cells (HSPCs) were obtained by using EasySep^™^ Mouse Hematopoietic Progenitor Cell Isolation Kit (STEMCELL Technologies, Vancouver, Canada). HSPCs were cultured in expansion medium (STEMCELL Technologies, Vancouver, Canada) with 100 ng/mL SCF (PeproTech, NJ, USA) and 20 ng/mL TPO (PeproTech, NJ, USA) for 5 days. Cells were collected after cultured for 5 day and seeded in StemSpan^™^ serum-free expansion medium containing 20 ng/mL TPO alone. Cultures were continued for an additional 7 days to generate mature MKs.

### Cell counting Kit-8 (CCK-8) assay

Cell proliferation of K562 and Meg-01 cells was evaluated using a CCK-8 Kit (APExBIO, Houston, TX, USA. Briefly, K562 and Meg-01 cells were treated with different concentrations of RR (5, 10, and 20 μM) or emodin (2.5, 5, and 10 μM) for 1, 3, or 5 days. CCK-8 reagent was added at the indicated time points, and cells were incubated at 37 °C for 1 h prior to detection of absorbance at 450 nm.

### Lactate dehydrogenase (LDH) assay

Cytotoxicity was assessed by measuring LDH release using a commercial LDH assay Kit. K562 and Meg-01 cells were treated with RR (5, 10, and 20 μM) or emodin (2.5, 5, and 10 μM) for 1, 3, or 5 days. At each time point, LDH working solution (Beyotime, Jiangsu, China) was added. Absorbance values were obtained at 490 nm.

### Morphological observation

K562, Meg-01, and HSPCs were treated with RR granules (5, 10, and 20 μM) or emodin (2.5, 5, and 10 μM), or PMA (1 nM) for 5 days. Cell morphology was observed and recorded using an inverted phase-contrast microscop.

### Giemsa staining

Cells were treated with RR (5, 10, and 20 μM), or emodin (2.5, 5, and 10 μM) for 5 days. After collection, cells were subjected to fixation using a methanol/acetic acid mixture at a ratio of 3:1 for 5 min. Fixed cells were incubated with a mixed Giemsa working solution (Solarbio, Beijing, China) for 8 min. After washing with PBS, slides were examined and photographed under a Nikon light microscope.

### Immunofluorescence

Cells were fixed in 4% paraformaldehyde for 10 min and permeabilized with 0.5% Triton X-100 for 5 min. Samples were blocked with 5% bovine serum albumin (BSA) at room temperature for 1 h and then incubated with primary antibodies including RUNX1 (Proteintech, 25,315-1-AP), EGR1 (Proteintech, 22,008-2-AP), or NF-E2 (Proteintech, 11,089-1-AP). After thorough washing with PBS, slides were incubated with appropriate fluorescent secondary antibodies. Nuclei were briefly labeled with DAPI (30 s) and visualized under fluorescent illumination.

### Flow cytometric analysis of MK differentiation *in vitro*

MK differentiation was examined in both cell-line models and primary mouse HSPCs. For cell-line assays, a 100-μL aliquot of each sample was incubated with 7 μL FITC-CD41 and 5 μL PE-CD42b (Beijing 4A Biotech, Beijing, China) for 20 min on ice in the dark. For primary assays, HSPCs were collected and washed, followed by stepwise centrifugation to enrich MKs and platelets: 1200 rpm for 5 min to pellet MKs, and 3000 rpm for 10 min to pellet platelets. After resuspension, 100 μL of each sample was incubated with 0.5 μL FITC-CD41 (BioLegend, San Diego, CA, USA) and 2.5 μL PerCP-Cy5.5-CD61 (BioLegend, San Diego, CA, USA). All samples were analyzed on a flow cytometer. Data were processed using FlowJo software.

### Flow cytometric analysis of MK polyploidization *in vitro*

The treated cells were fixed with 70% ethanol and incubated with PI/RNase staining buffer (Beyotime, Jiangsu, China) on ice for 15 min. Flow cytometry was employed to determine cellular DNA content.

### Apoptosis assay

Apoptosis was evaluated by Annexin V-FITC/PI Apoptosis Detection Kit (Vazyme, Nanjing, China) according to the manufacturer’s protocol.

### Establishment of a mouse model of thrombocytopenia and drug treatment

Two parallel sets of in vivo studies were performed to assess thrombopoietic activities of RR and emodin. Male Kunming (KM) mice (6–8 weeks old, 18–22 g) were used in all in vivo experiments to minimize potential variability associated with estrous-cycle-dependent hormonal fluctuations. For the RR study, mice were divided into the following groups (n = 8 per group): normal control, model, rhTPO (3000 U/kg) positive control, and RR (20, 40, and 80 mg/kg) groups. For the emodin study, mice were assigned to normal control, model, rhTPO positive control, and emodin (2.5, 5, and 10 mg/kg) groups. Except for the normal groups, all mice received a single 4 Gy dose of whole-body irradiation. The normal and model groups received daily intraperitoneal injections of physiological saline. rhTPO, RR or emodin groups received the corresponding test compound by intraperitoneal injection at the designated doses once daily for 14 consecutive days. All animal experiments were approved by the Animal Ethics Committee of Southwest Medical University (approval number: 20240728-002).

### Hematological analysis

On days 0, 3, 7, 10, and 12 of treatment, approximately 40 μL of blood was obtained from retro-orbital venous plexus and incubated with 160 μL of diluent for hematological analysis. Platelet, white blood cells (WBC), and red blood cells (RBC) were counted using automated hematology analyze.

### Histological analysis

On day 12, femurs, liver, spleen, heart, lung, kidney, and thymus were collected and fixed in 4% paraformaldehyde for at least 24 h. After prolonged decalcification of the femurs, tissues were processed for paraffin embedding. Serial Sects (5 μm) were prepared and stained using hematoxylin and eosin (H&E).

### Organ index

After 12 days of treatment, heart, liver, spleen, lung, kidneys, and thymus were excised, blotted dry, and weighed. Organ indices were expressed as organ weight normalized to body weight (mg/g).

### Assessment of serum biochemical parameters

Serum concentrations of alanine aminotransferase (ALT), aspartate aminotransferase (AST), UREA, LDH, and creatine kinase (CK) were measured using an automated biochemical analyzer.

### Flow cytometric analysis of BM, lung, and spleen cells

Spleen and lung tissues were gently homogenized to obtain single-cell suspensions, which were passed through a 200-mesh nylon filter. Cells were counted using a hematology analyzer and adjusted to 1 × 10^6^ cells/mL. BM suspension was incubated with combinations of fluorophore-conjugated antibodies, including FITC-CD41, PE-CD61, APC-CD42, PE-CD117 (c-Kit), FITC-CD34, and PI. Spleen and lung samples were stained with FITC-CD41, PE-CD61 and PI, and then analyzed using flow cytometry.

### Network pharmacology analysis of RR

Twenty RR-derived constituents identified by UHPLC–HRMS were searched in the PubChem database to obtain their SMILES structures. Each SMILES was imported into the SwissTargetPrediction database to predict putative protein targets, and the resulting names were standardized to official gene symbols by UniProt database. Thrombocytopenia-related genes were collected from GeneCards, OMIM, and the Therapeutic Target Database (TTD), after which duplicate entries were removed. The predicted RR targets were compared with thrombocytopenia-associated genes, and overlapping targets were identified using Venn diagram tool. Protein–protein interaction (PPI) networks for overlapping targets were constructed using STRING database (species set to Homo sapiens; confidence threshold = 0.900). Nodes without interactions were hidden, and the interaction file was exported in TSV format. The network was visualized in Cytoscape 3.10.0, and hub genes were identified based on node degree. For clarity of visualization, subnetworks were generated using degree cutoffs of > 89, > 46, and > 22. RR-target-disease interaction networks were built in Cytoscape to explore the relationships between RR constituents, core targets, and thrombocytopenia. Functional enrichment analyses were conducted by importing overlapping genes into the DAVID database for Gene Ontology (GO) annotation and Kyoto Encyclopedia of Genes and Genomes (KEGG) pathway enrichment. Pathways with *p* < 0.05 were considered significant, and bubble plots were generated according to adjusted *p*-values and fold enrichment.

### Network pharmacology analysis of emodin

The chemical structure of emodin was retrieved from PubChem and imported into SwissTargetPrediction to predict potential targets. Emodin-related targets were also obtained from TCMSP database, and all protein names were standardized to gene symbols using UniProt. The union of targets from the two databases was used for subsequent analyses. Genes associated with MK differentiation were obtained from GeneCards database using keyword “megakaryocyte differentiation,” and thrombocytopenia-related genes were obtained from GeneCards, OMIM, and TTD. After removing duplicates, emodin targets were intersected separately with MK differentiation genes and thrombocytopenia genes using Venn analysis to obtain shared targets relevant to both processes. Overlapping genes from emodin vs. MK differentiation and emodin vs. thrombocytopenia comparisons were imported into STRING (confidence = 0.400; species = Homo sapiens) to construct independent PPI networks. Networks were downloaded in TSV format and visualized in Cytoscape 3.10.0. Core regulatory nodes were selected according to degree values (> 10). Comprehensive emodin-target-MK differentiation–thrombocytopenia interaction networks were subsequently established using Cytoscape to illustrate relationships among emodin, predicted targets, and disease-relevant biological processes. For functional interpretation, overlapping genes were submitted to the DAVID database for GO and KEGG enrichment analyses. Pathways with statistical significance (*p* < 0.05) were included in downstream visualization using bubble plots.

### RNA sequencing

Meg-01 cells were treated with RR (20 μg/mL) or emodin (10 μM) for 3 days, after which total RNA was extracted. High-quality RNA samples were used for library construction. Sequencing libraries were generated by Illumina stranded mRNA library preparation workflow by Shanghai Majorbio Bio-Pharm Technology Co., Ltd. Raw reads were processed for quality control using fastp, and clean reads were aligned to the human reference genome using HISAT2. Differentially expressed genes (DEGs) were identified with fold-change ≥ 1.5 and *p* < 0.05 as significance thresholds. GO and KEGG enrichment analyses of differentially expressed genes were performed using GOATOOLS and KOBAS, respectively.

### Molecular docking

The X-ray crystal structure of ESR1 (PDB: 8DV7) was obtained from Protein Data Bank. The protonation state of the small-molecule ligand was adjusted to pH 7.4, and its 3D structure was generated using Open Babel. Receptor and ligand preparation was performed using AutoDockTools (ADT 3). The docking grid box was constructed using AutoGrid, followed by molecular docking with AutoDock Vina (version 1.2.0), and visualized and rendered using PyMOL.

### Molecular docking stimulation

Molecular dynamics simulations were conducted using the Desmond/Maestro non-commercial software package (version 2022.1). The protein-ligand complex was solvated in an orthorhombic box using the TIP3P water model, and 0.15 M NaCl was added to neutralize and equilibrate the system. After energy minimization and relaxation of the solvated complex, a 100-ns production molecular dynamics simulation was carried out under an isothermal–isobaric ensemble at 300 K and 1 bar. Trajectory coordinates were recorded every 100 ps. Post-simulation analyses, including root mean square deviation (RMSD), root mean square fluctuation (RMSF), hydrogen-bond interactions, and protein–ligand contact profiles, were performed using Simulation Interaction Diagram module in Desmond.

### ESR1 siRNA transfection

ESR1 knockdown was performed using small interfering RNA (siRNA) transfection in Meg-01 cells. Cells were collected by centrifugation, washed once with PBS, and resuspended in complete culture medium. After cell counting, cells were seeded into 12-well plates at a density of approximately 3 × 10^5^ cells per well in 1000 μL complete medium. Negative control (NC) siRNA and three ESR1-targeting siRNAs were purchased from GenePharma. One OD of each siRNA was dissolved in 125 μL DEPC-treated water to prepare a 20 μM stock solution. For each well, 1.25 μL of 20 μM siRNA was mixed with 1.25 μL siRNA-Mate SUS 2 × Buffer to obtain 2.5 μL siRNA–buffer mixture. Then, 2.5 μL siRNA-Mate SUS Reagent was added to the mixture to generate a final transfection complex volume of 5 μL. The complex was gently mixed by pipetting several times without vortexing and incubated at room temperature for 5 min. The transfection complex was then added directly to 1000 μL complete medium containing cells, resulting in a final siRNA concentration of 50 nM. Cells were cultured at 37 °C in a humidified atmosphere containing 5% CO_2_. On the third day after seeding, cells were collected for subsequent analyses. The siRNA sequences were as follows: NC siRNA, sense 5′-UUC UCC GAA CGU GUC ACG UTT-3′ and antisense 5′-ACG UGA CAC GUU CGG AGA ATT-3′; ESR1 siRNA1, sense 5′-CCA AUG ACA AGG GAA GUA UTT-3′ and antisense 5′-AUA CUU CCC UUG UCA UUG GTT-3′; ESR1 siRNA2, sense 5′-CAG GCU ACC AUU AUG GAG UTT-3′ and antisense 5′-ACU CCA UAA UGG UAG CCU GTT-3′; ESR1 siRNA3, sense 5′-GAU ACU CUA UUC CGA GUA UTT-3′ and antisense 5′-AUA CUC GGA AUA GAG UAU CTT-3′.

### Western blot analysis

Following cell lysis in RIPA buffer, total protein was collected and equalized prior to electrophoretic separation on 10% SDS-polyacrylamide gels. The resolved proteins were transferred to PVDF membranes, blocked under room temperature conditions, and probed with primary antibodies including ESR1 (Proteintech, IL, USA, 20698-1-AP), p-JAK2 (Abmart, T56570s), JAK2 (Abmart, T55287s), p-STAT3 (Abmart, T56566s), STAT3 (Abmart, T55292s), p-PI3K (Abmart, T40116s), PI3K (Abmart, PA4491s), p-AKT (Abmart, T40067s), AKT (Abmart, T55561s), RUNX1 (Proteintech, 25315-1-AP), EGR1 (Proteintech, 22008-2-AP), NF-E2 (Proteintech, 11089-1-AP), and GAPDH (Elabscience, E-AB-40337). Membranes were then treated with secondary antibodies and visualized through ECL detection Kit.

### Cellular thermal shift assay (CETSA)

Cell lysates were preincubated with emodin (2.5, 5, or 10 μM) or DMSO for 1 h. Aliquots were then heated at 37, 42, 47, 52, or 57 °C for 10 min, cooling on ice and centrifugation to remove precipitated proteins. Supernatants were collected and ESR1 levels were analyzed by western blot to assess changes in thermal stability [24].

### Drug affinity responsive target stability (DARTS) assay

Cell lysates were incubated with emodin (2.5, 5, or 10 μM) for 1 h at room temperature. Samples were then treated with different dilutions of pronase E (1:1000, 1:1500, or 1:3000) and incubated at 40 °C for 10 min. Protease digestion was terminated by adding SDS sample buffer, and ESR1 levels were assessed by western blot as described above [24, 25].

### Statistical analysis

Experimental procedures were independently conducted no fewer than three times. Data are reported as mean values with corresponding standard deviation (SD). For comparisons among multiple groups, one-way ANOVA was used. For time-course experiments or analyses involving two independent variables, two-way ANOVA was applied. Statistical differences were considered significant when *p* values were below 0.05.

## Results

### Chemical characterization of RR

**Fig. 1:**
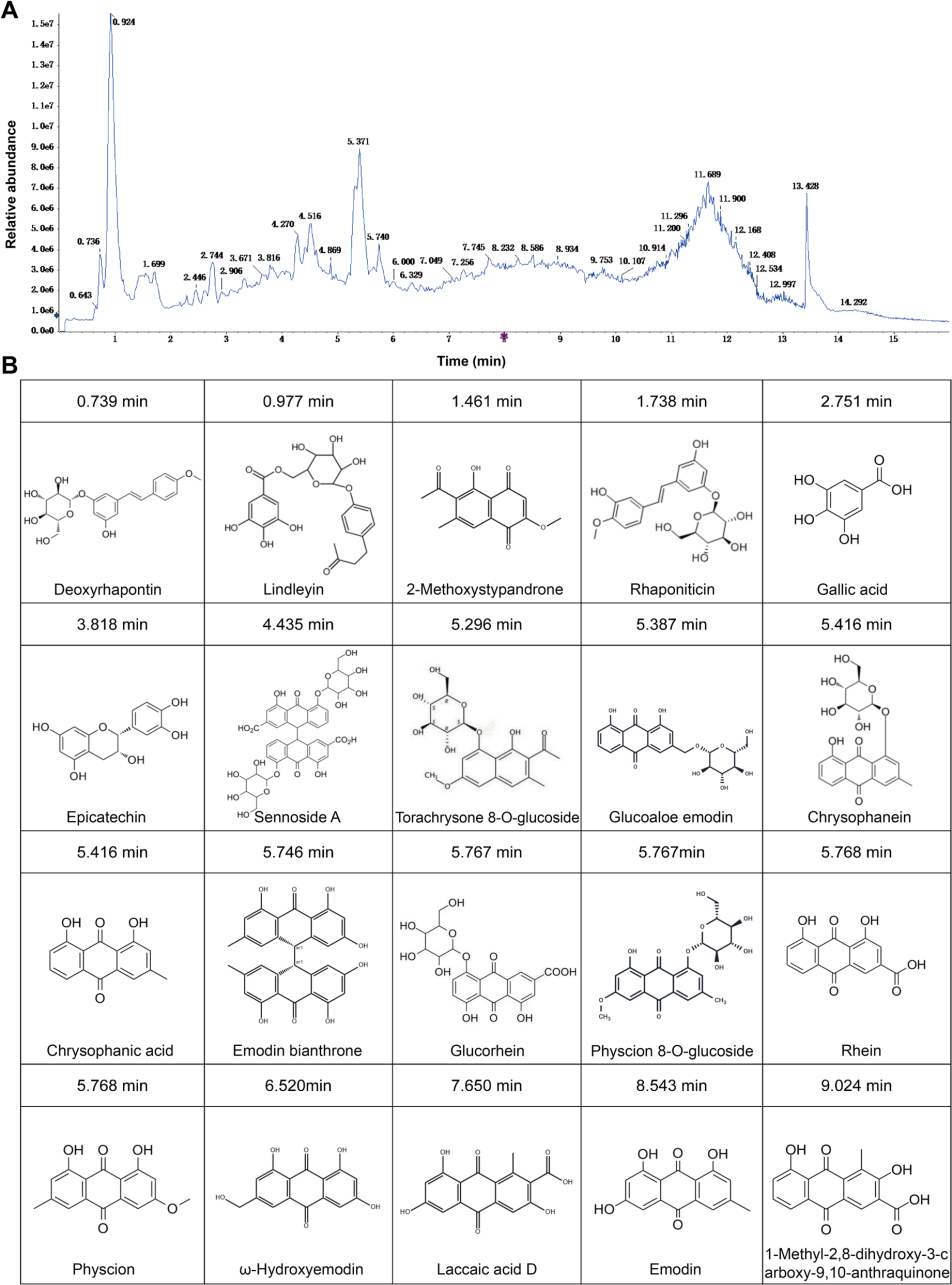
Chemical characterization of the RR. **A** TIC of the RR analyzed by UHPLC–HRMS. The complex chemical profile is demonstrated, with major chromatographic peaks labeled by their retention times (min). **B** Representative chemical structures and corresponding retention times (min) of the compounds identified in the RR

To elucidate the chemical basis underlying the thrombopoietic activity of RR, we first performed a systematic profiling of the aqueous extract using UHPLC-HRMS. The total ion chromatogram (TIC) revealed a dense pattern of chromatographic peaks within a short analytical window (Fig. 1A), indicating the presence of abundant and structurally diverse metabolites. Based on accurate mass measurements, MS/MS fragmentation analysis, and comparison with published reference spectra, more than twenty constituents were identified (Fig. 1B). The identified compounds were primarily distributed across several phytochemical group characteristic of RR, including anthraquinones, polyphenols, and phenylethanoid glycosides. Anthraquinone derivatives such as rhein, emodin, physcion, and their glycosylated forms were clearly detected and represented the major chemical scaffold of the extract. In addition, several phenolic acids (e.g., gallic acid) and flavan-3-ols (e.g., epicatechin) were observed (Fig. 1), further illustrating the phytochemical complexity of RR. Notably, emodin—the principal active molecule examined in subsequent experiments—was identified with a distinct retention time of 8.543 min. To further quantify emodin in the RR extract, LC–MS/MS analysis was performed using an emodin standard calibration curve. The calibration curve showed good linearity over the tested concentration range, with a regression equation of y = 35.8169*x* − 1925.8497 and an R^2^ value of 0.997. Based on this quantitative analysis, the emodin content in RR was determined to be 0.44 mg/g (Fig. S1). This comprehensive chemical characterization provided a rigorous foundation for subsequent pharmacological validation and guided our efforts to link the constituents of RR with its thrombopoietic effects.

### *RR promotes MK differentiation of *in vitro

**Fig. 2:**
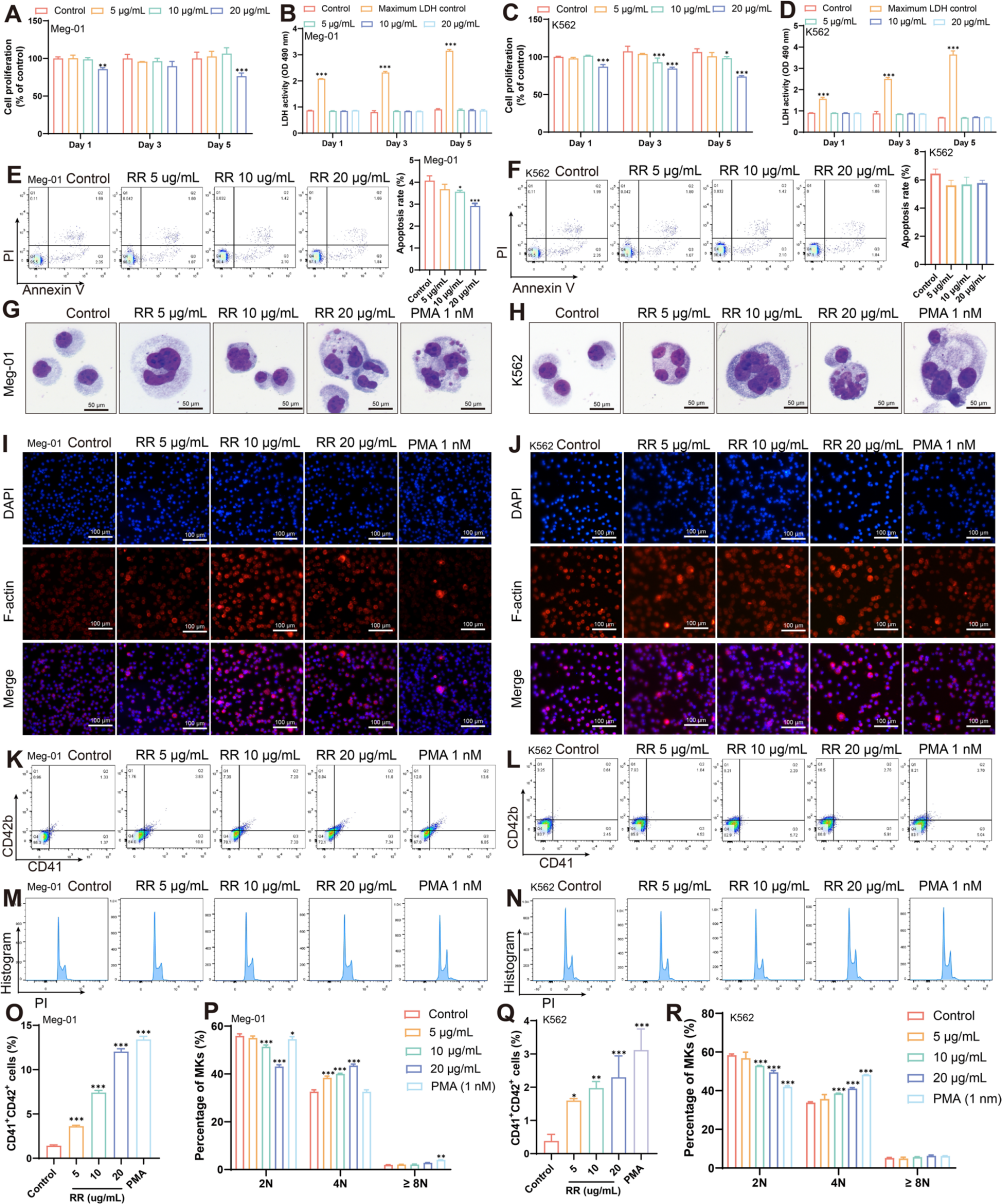
RR facilitates MK differentiation in vitro. **A**–**D** Effects of RR on cell viability and cytotoxicity. Meg-01 **A**, **B** and K562 **C**, **D** cells were treated with RR (5, 10, 20 μM) for 1, 3, and 5 days. Cell proliferation was assessed by CCK-8 assay **A**, **C** (n = 3). LDH release was measured to evaluate membrane integrity and cytotoxicity **B**, **D**. Maximum LDH served as a positive control (n = 3). **E**, **F** Apoptosis analysis by Annexin V/PI staining in Meg-01 **E** and K562 **F** cells (n = 3). **G**, **H** Giemsa staining of Meg-01 **G** and K562 **H** cells after 5-day treatment with RR or PMA (1 nM). Scale bar = 50 μm. **I**, **J** Phalloidin staining showing cytoskeletal remodeling and multinucleated cell formation in Meg-01 **I** and K562 **J** cells following RR treatment. F-actin (red) and nuclei (DAPI, blue) are shown. Scale bar = 100 μm. **K**, **L** Flow cytometric analysis of CD41 and CD42b in Meg-01 K and K562 **L** cells treated with RR or PMA for 5 days (n = 3). **M**, **N** DNA ploidy distribution in Meg-01 M and K562 **N** cells after RR treatment (n = 3). **O**–**R** Quantitative analysis of the percentage of CD41⁺CD42b⁺ cells in Meg-01 **O** and K562 **P**, and DNA ploidy in Meg-01 **Q** and K562 **R** (n = 3). For A-D, P and R, statistical significance was determined using two-way ANOVA; for E–F, O and Q, one-way ANOVA was used. *p < 0.05, **p < 0.01, ***p < 0.001 vs. corresponding control

Following the chemical characterization of the RR, we next examined its biological activity in vitro, beginning with an evaluation of cellular safety in Meg-01 and K562 cells. CCK-8 analysis showed that RR maintained cell viability over a 1–5-day treatment period, with only a slight reduction observed at the highest concentration tested (Fig. 2A, C). LDH measurements further supported the absence of cytotoxicity, as LDH release remained unchanged relative to the control group throughout the observation period (Fig. 2B, D). Flow cytometric analysis of apoptosis provided additional confirmation of this safety profile, revealing that RR reduced the proportion of apoptotic Meg-01 cells and did not induce apoptosis in K562 cells (Fig. 2Q, R). These findings collectively indicate that RR is well-tolerated within the effective concentration range. With cellular safety established, we next assessed whether RR could influence MK differentiation. Morphological assessment by giemsa staining showed that RR (5, 10, and 20 μg/mL) induced prominent features of MK maturation in both Meg-01 and K562 cells, resembling the effects of positive control PMA (1 nM). Treated cells displayed enlarged, lobulated nuclei—an established marker of MK differentiation (Fig. 2E, F). Consistent with these observations, phalloidin staining demonstrated marked cytoskeletal remodeling and the presence of multinucleated cells, reflecting advancing cytoplasmic maturation (Fig. 2G, H). Flow cytometric quantification of differentiation markers further substantiated these results. RR increased the proportion of CD41⁺CD42b⁺ cells in a concentration-dependent manner in both cell lines (Fig. 2I, J, M, O), indicating enhanced megakaryocytic lineage commitment. In parallel, RR promoted polyploidization—a defining step of MK maturation—evidenced by a reduction in the 2N population and a corresponding rise in 4N cells (Fig. 2K, L, N, P). Taken together, these findings demonstrate that RR is not only safe in vitro but effectively drives multiple hallmarks of MK differentiation.

### RR stimulates multi-compartmental megakaryopoiesis leading to enhanced platelet-producing acapacity in RIT mice

**Fig. 3:**
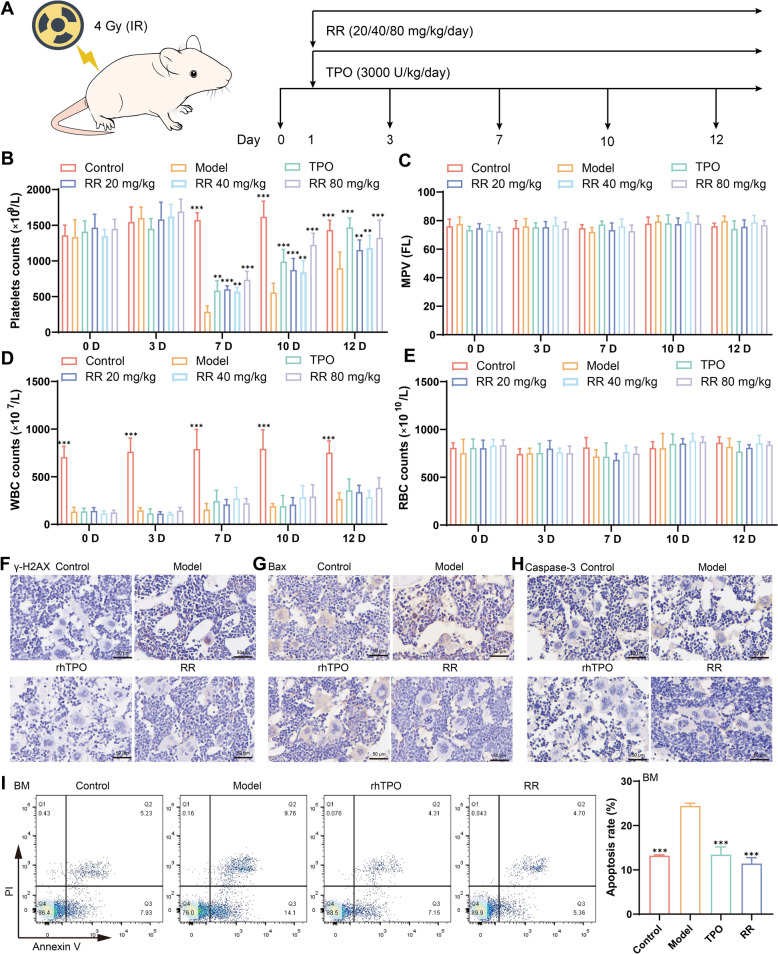
RR promotes platelet regeneration and alleviates radiation-induced BM injury in RIT mice. **A** Schematic illustration of the experimental procedure for establishing the RIT model and the subsequent administration of RR or rhTPO. **B-E** Time-course analysis of peripheral platelet counts **B**, MPV **C**, WBC **D** and RBC counts **E** following irradiation and daily administration of RR (20, 40, and 80 mg/kg) or rhTPO for 12 days (n = 8). **F**–**H** Immunohistochemical staining of γ-H2AX **F**, Bax **G**, and Caspase-3 **H**. Scale bar = 50 μm. **I** Flow cytometric assessment of BM apoptosis using Annexin V/PI staining (n = 3). Data are presented as mean ± SD. For B-E, statistical significance was determined using two-way ANOVA; for I, one-way ANOVA was used. *p < 0.05, **p < 0.01, ***p < 0.001 vs. model group

Having established the in vitro thrombopoietic activity of RR, we proceeded to evaluate its efficacy in RIT mice. RIT mice were treated daily with RR (20, 40, and 80 mg/kg) or rhTPO (3000 U/kg) for 12 consecutive days (Fig. 3A). Peripheral blood analysis demonstrated that platelet counts in the model group decreased markedly, with the most pronounced reduction occurring on day 7, followed by a spontaneous recovery. Both RR and rhTPO markedly mitigated the radiation-induced decrease in platelet levels at day 7, and significantly accelerated platelet recovery at days 10 and 12 (Fig. 3B). Mean platelet volume (MPV) remained unchanged (Fig. 3C), suggesting that RR primarily enhanced platelet production rather than altering platelet size. WBCs exhibited the expected radiation-induced reduction and gradual recovery; RR produced a modest but non-significant improvement (Fig. 3D). RBC levels were not affected by RR treatment (Fig. 3E). These findings indicate that RR exerts a selective thrombopoietic effect under RIT conditions. We further examined the extent of BM injury after irradiation. Immunohistochemical staining for γ-H2AX revealed a pronounced increase in γ-H2AX-positive cells in BM of irradiated mice, whereas treatment with RR or rhTPO significantly reduced γ-H2AX expression (Fig. 3F), indicating attenuation of radiation-induced DNA damage. Radiation also induced upregulation of Bax and Caspase-3 in BM tissue—two key mediators of apoptosis. Both markers were markedly decreased in mice treated with RR or rhTPO (Fig. 3G, H), suggesting that RR alleviates irradiation-induced apoptotic injury. Flow cytometric analysis of BM cells corroborated these observations: the proportion of apoptotic cells was significantly higher in the irradiated group than in controls, whereas RR and rhTPO treatment substantially lowered apoptosis rates. Together, these data demonstrate that RR not only promotes platelet regeneration in vivo but also provides meaningful protection against radiation-induced bone marrow damage by reducing DNA injury and apoptosis.

**Fig. 4:**
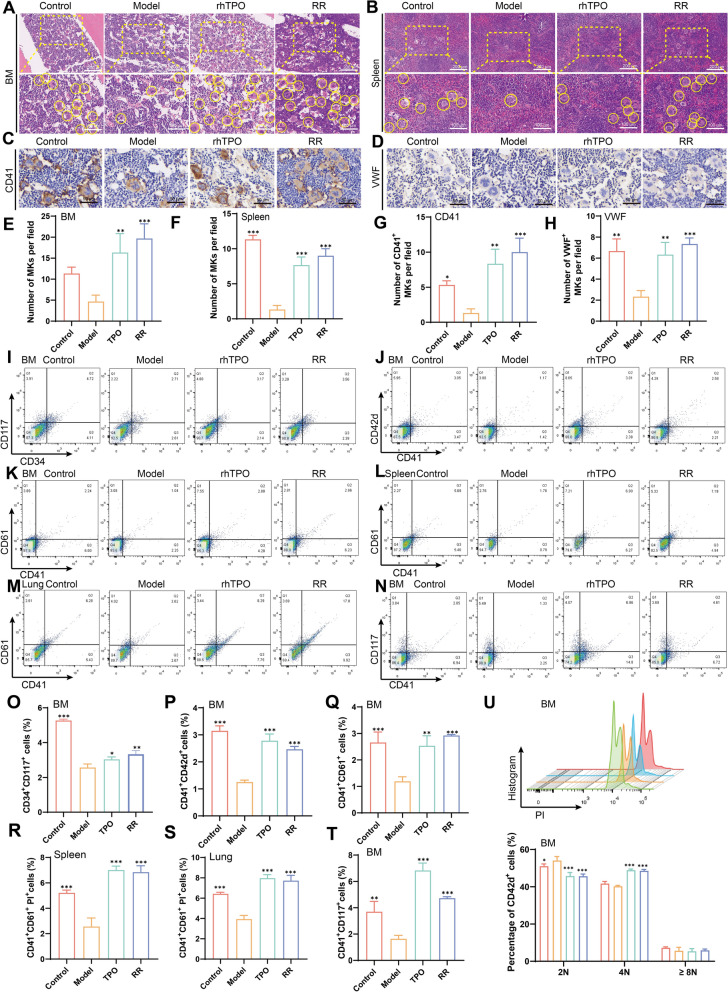
RR promotes megakaryopoiesis and terminal differentiation across multiple hematopoietic compartments in RIT mice. **A**–**B** H&E staining of BM **A** and spleen **B** sections from Control, Model, rhTPO, and RR-treated mice. Representative low- and high-magnification images are shown. MKs are indicated by yellow circles. Scale bars = 200 μm (upper panels), 100 μm (lower panels). **C**–**D** Immunohistochemical staining of BM sections for CD41 **C** and VWF **D**. Representative images and corresponding quantitative analyses of CD41⁺ and VWF⁺ MKs per field. Scale bars = 50 μm. **E**–**H** Quantification of MKs per field in BM **E**, spleen **F**, CD41⁺ MKs **G**, and VWF⁺ MKs **H**. **I**–**N** Flow cytometric analyses of the proportions of CD34⁺CD117⁺ cells in BM **I**, CD41⁺CD117⁺ cells in BM **J**, CD41⁺CD42d⁺ cells in BM **K**, CD41⁺CD61⁺ cells in BM **L**, CD41⁺CD61⁺PI^+^ cells in spleen **M**, and CD41⁺CD61⁺PI^+^ cells in lung **N** (n = 3). **O** DNA ploidy distribution of BM CD42d⁺ MKs assessed by PI staining (n = 3). **P**–**U** Quantitative analyses corresponding to CD34⁺CD117⁺ cells in BM **P**, CD41⁺CD117⁺ cells in BM **Q**, CD41⁺CD42d⁺ cells in BM **R**, CD41⁺CD61⁺ cells in BM **S**, CD41⁺CD61⁺PI^+^ cells in spleen **T**, and CD41⁺CD61⁺PI^+^ cells in lung **U** (n = 3). Data are presented as mean ± SD. Statistical significance was determined using one-way ANOVA; for U, two-way ANOVA was used. *p < 0.05, **p < 0.01, ***p < 0.001 vs. model group

Given that peripheral platelet recovery is tightly linked to MK output, we next examined whether the thrombopoietic effect of RR in vivo was associated with enhanced MK biogenesis. H&E staining of BM sections showed that irradiation markedly reduced number of MKs, consistent with radiation-induced hematopoietic suppression (Fig. 4A). Treatment with RR or rhTPO substantially increased MK counts relative to the model group (Fig. 4A), indicating a robust restorative effect on BM megakaryopoiesis. Because the spleen serves as an auxiliary extramedullary hematopoietic organ capable of MK expansion under stress conditions [26], we evaluated splenic MKs as well. Similar to the BM, RR and rhTPO administration significantly increased splenic MK numbers compared with irradiated models (Fig. 4B), suggesting coordinated enhancement of systemic MK production. To further verify MK identity and quantify lineage-specific restoration, immunohistochemical staining for the megakaryocytic markers CD41 and VWF was performed [27]. Irradiation decreased CD41⁺ and VWF⁺ MKs, whereas RR and rhTPO treatment restored their numbers to levels markedly higher than model group (Fig. 4C, D). These findings confirm that RR promotes the regeneration of functional MKs within the BM microenvironment. Because MKs arise from hematopoietic stem and progenitor cells (HSPCs), we next assessed whether RR influenced early hematopoietic differentiation. Flow cytometric analysis demonstrated that RR and rhTPO markedly increased the proportion of CD34⁺CD117⁺ HSPCs, as well as CD117⁺CD41⁺ megakaryocytic progenitors in the BM (Fig. 4I, J, P, Q). These results indicate that RR accelerates the progression of HSPCs toward the megakaryocytic lineage and expands the intermediate MK progenitor pool. We further examined the maturation status of MKs, as only fully differentiated MKs can release platelets. RR and rhTPO significantly elevated the proportion of CD41⁺CD42d⁺ and CD41⁺CD61⁺ MKs in the BM (Fig. 4K, L, R, S), indicating enhanced terminal differentiation. Similar effects were observed in the spleen, where RR increased CD41⁺CD61⁺PI⁺ MKs (Fig. 4M, T), suggesting active extramedullary MK maturation. The lung, a major site of platelet biogenesis, displayed the same trend: RR and rhTPO treatment increased CD41⁺CD61⁺PI⁺ MKs (Fig. 4N, U), supporting improved pulmonary MK differentiation. Finally, analysis of DNA ploidy revealed that RR and rhTPO reduced the proportion of 2N CD42d⁺ MKs but increased the proportion of 4N CD42d⁺ MKs (Fig. 4O), demonstrating that RR effectively promotes endomitosis, a critical step for MK maturation and subsequent platelet release. Collectively, these data establish that RR drives robust thrombopoiesis by enhancing megakaryocytic lineage commitment, proliferation, differentiation, and terminal maturation across multiple hematopoietic compartments.

### Integrated network pharmacology and transcriptomic analysis reveal RR activation of megakaryocytic and thrombopoietic pathways

**Fig. 5:**
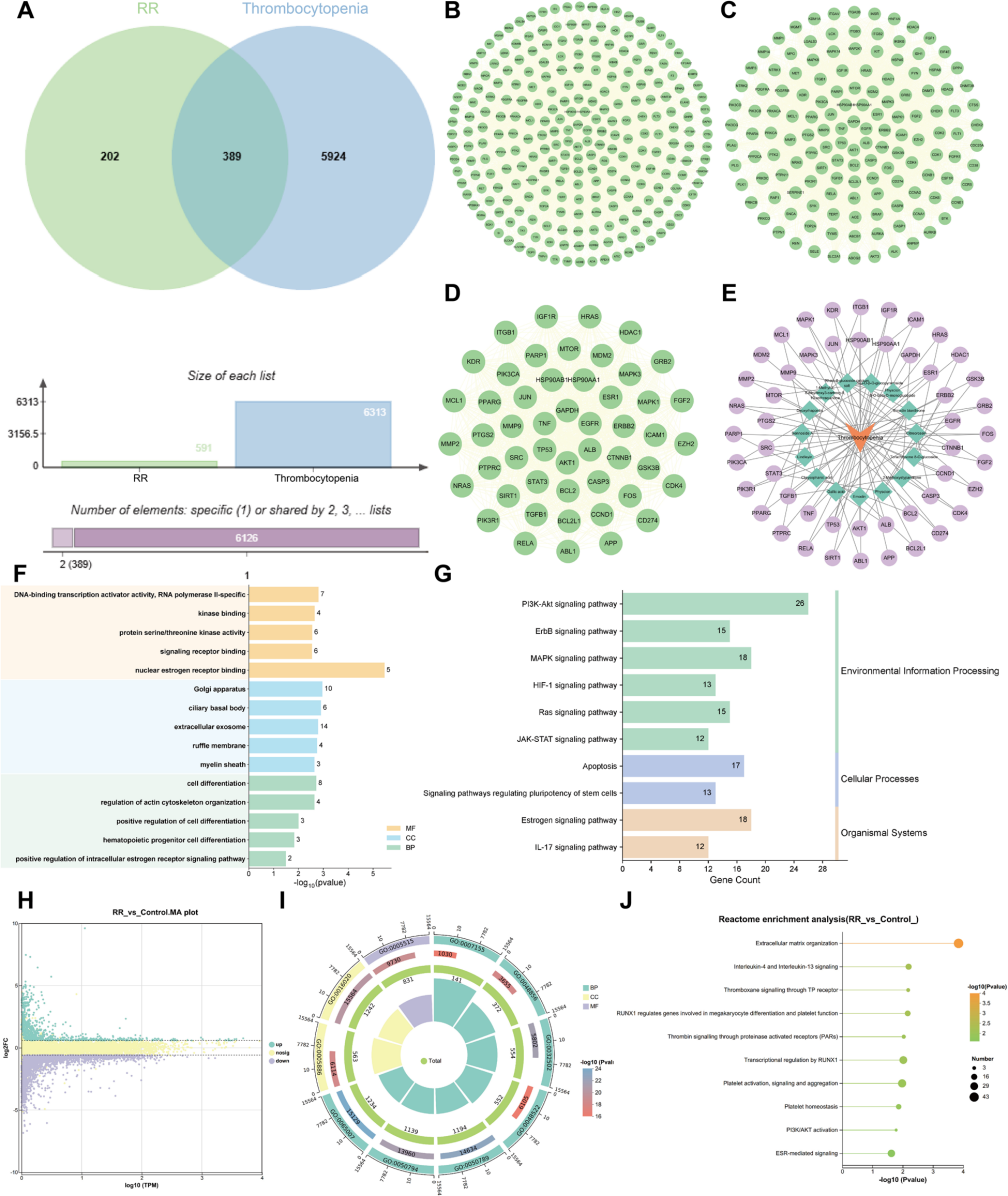
Network pharmacology and transcriptomic analysis reveal the putative targets and signaling pathways of RR against thrombocytopenia. **A** Venn diagram showing the overlap between predicted RR-related targets and thrombocytopenia-associated gene. **B**–**D** PPI networks of overlapping targets constructed using STRING (confidence = 0.900) and visualized in Cytoscape. Subnetworks were generated according to node degree thresholds (> 89 in B, > 46 in C, and > 22 in D). **E** RR constituent–target–disease interaction network illustrating the relationships among identified RR compounds, their predicted targets, and thrombocytopenia-associated genes. **F** GO functional enrichment analysis (Biological Process (BP), Cellular Component (CC), Molecular Function (MF) categories) of overlapping genes. **G** KEGG pathway enrichment analysis of overlapping targets. **H** MA plot of DEGs in RR-treated Meg-01 cells. **I** GO enrichment analysis of RR-regulated genes. **J** Reactome pathway enrichment analysis of RR-responsive DEG

To clarify molecular basis of thrombopoietic ability of RR, we first performed a network pharmacology analysis integrating RR-derived constituents with thrombocytopenia-associated gene datasets. Among the 20 RR components identified by UHPLC–HRMS, target prediction via SwissTargetPrediction yielded 591 putative protein targets, while 6313 thrombocytopenia-related genes were retrieved from GeneCards, OMIM, and TTD. A Venn analysis demonstrated 389 common genes shared between RR-related targets and thrombocytopenia-associated genes (Fig. 5A), indicating a substantial molecular intersection relevant to platelet biogenesis. To characterize the functional architecture of these overlapping genes, a high-confidence PPI network was generated using STRING (confidence score ≥ 0.900). The full network revealed extensive protein connectivity (Fig. 5B), and progressive degree-based filtering (thresholds > 89, > 46, and > 22) facilitated the identification of key regulatory hubs (Fig. 5C, D). Notably, AKT1, MAPK1, STAT3, ESR1, EGFR, JUN, and SRC emerged as major nodes with high centrality—genes well-established as critical regulators in hematopoietic proliferation, MK maturation, and platelet production[28, 29]. Construction of the RR–component–target–disease network further demonstrated that multiple RR components converged on these central signaling molecules (Fig. 5E), suggesting a coordinated, multi-target mode of action consistent with traditional herbal pharmacology. Functional enrichment analyses reinforced these observations. GO analysis highlighted biological processes linked to cytoskeletal remodeling, cellular differentiation, stem/progenitor cell regulation, and positive regulation of estrogen receptor signaling (Fig. 5F). KEGG analysis showed significant enrichment of pathways intimately involved in MK differentiation and platelet generation, including PI3K–AKT, MAPK, RAS, JAK–STAT, HIF-1, and estrogen signaling (Fig. 5G). These findings strongly suggested that RR may promote MK development and platelet production through simultaneous engagement of multiple pathways.

To experimentally substantiate the mechanisms predicted through network pharmacology, RNA sequencing on Meg-01 cells treated with RR for 3 days was conducted. Differential expression analysis identified a substantial set of RR-responsive genes, indicating broad transcriptional remodeling (Fig. 5H). GO enrichment analysis revealed that RR-responsive genes were involved in biological regulation, regulation of cellular and developmental processes, cell adhesion, and membrane/plasma membrane–associated functions, indicating broad transcriptional activation of programs essential for megakaryocytic commitment, cytoskeletal remodeling, and cell–matrix interactions (Fig. 5I). Reactome analysis further refined these regulatory patterns and revealed a distinct clustering of pathways directly linked to thrombopoiesis. RR significantly enriched RUNX1-related modules, including “RUNX1 regulates genes involved in megakaryocyte differentiation and platelet function” and “Transcriptional regulation by RUNX1”, together with key platelet-associated pathways such as “Platelet activation, signaling and aggregation”, “Platelet homeostasis”, “Thromboxane signaling through TP receptor”, and “Thrombin signaling through PARs”. Additional enrichment in PI3K/AKT activation, ESR-mediated signaling, and IL-4/IL-13 signaling further supports the involvement of proliferative, hormonal, and inflammatory axes in RR-driven megakaryocytic maturation (Fig. 5J). When integrated with the network pharmacology findings-which identified 389 overlapping RR–thrombocytopenia targets and highlighted PI3K–Akt, MAPK, JAK–STAT, ESR1, and RUNX1 as central regulatory nodes-the transcriptomic data collectively demonstrate that RR activates a coordinated thrombopoietic program. These results place RR as a multi-target modulator that promotes megakaryocytic lineage commitment, functional maturation, and platelet-related transcriptional activity through convergent activation of RUNX1-governed differentiation pathways, platelet signaling modules, and PI3K/AKT–ESR1 regulatory circuits.

### Emodin is the principal bioactive component of RR driving MK differentiation and platelet production

**Fig. 6:**
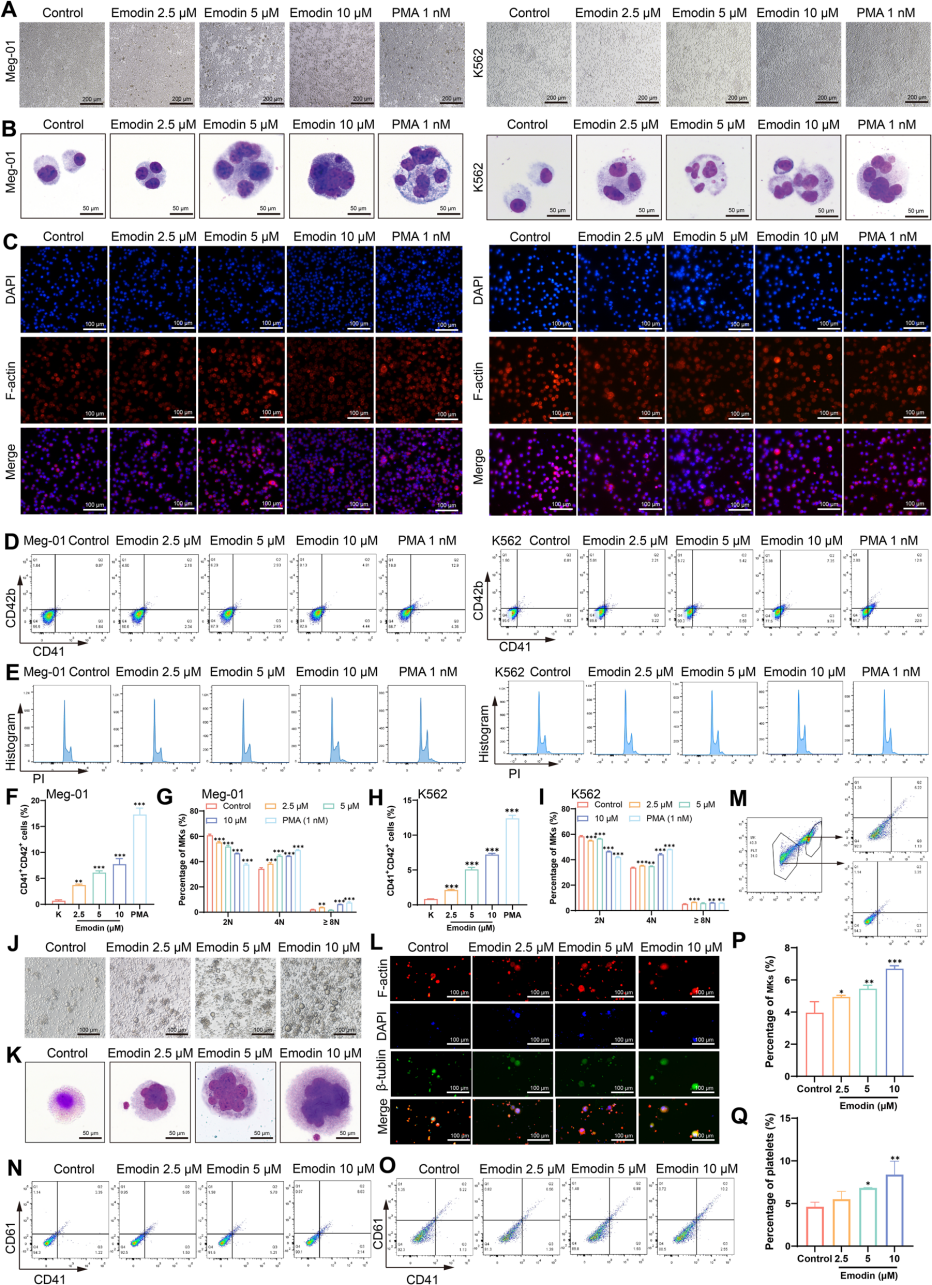
Emodin accelerates MK differentiation and platelet formation in Meg-01, K562, and primary murine HSPCs. **A** Representative phase-contrast images of Meg-01 and K562 cells treated with emodin (2.5, 5, and 10 μM) or PMA (1 nM). Scale bars = 200 μm. **B** Giemsa staining of Meg-01 and K562 cells treated with emodin. Scale bars = 50 μm. **C** Immunofluorescence staining for DAPI (nuclei) and F-actin in Meg-01 and K562 cells treated with emodin. Scale bars = 100 μm. **D** Flow cytometric analysis of CD41⁺CD42b⁺ MKs in Meg-01 and K562 cultures after emodin treatment (n = 3). **E** DNA ploidy distribution in Meg-01 and K562 cells assessed by PI staining (n = 3). **F**–**I** Quantification of CD41⁺CD42b⁺ MKs **F**, **H** and ploidy fractions **G**, **I** in Meg-01 and K562 cells (n = 3). **J** Phase-contrast images of primary murine HSPCs treated with emodin for 7 days. Scale bars = 100 μm. **K** Giemsa staining of HSPC after emodin treatment. Scale bars = 50 μm. **L** Immunofluorescence staining of HSPCs for DAPI, F-actin, and β-tubulin. Scale bars = 100 μm. **M**–**O** Flow cytometric identification of CD41⁺CD61⁺PI⁺ mature MKs **M**–**N** and CD41⁺CD61⁺PI⁻ platelets **O** derived from HSPCs (n = 3). **P**–**Q** Quantification of mature MKs **P** and platelets **Q** after emodin treatment (n = 3). For G and I, statistical significance was determined using two-way ANOVA; for F–H, P and Q, one-way ANOVA was used. *p < 0.05, **p < 0.01, ***p < 0.001 vs. control group

To identify the primary active component mediating the robust thrombopoietic effects of RR, eight representative RR-derived compounds identified by UHPLC–HRMS were subjected to an initial functional screening in Meg-01 cells. Morphological assessment (bright-field and Giemsa staining) and flow cytometric quantification of megakaryocytic markers (CD41 and CD42b) revealed that rhein, rhapontin, sennoside A, gallic acid, laccaic acid, and chrysophanol did not induce noticeable changes in either nuclear morphology or in the proportion of CD41⁺CD42b⁺ cells across concentrations from 5 to 20 μM (Fig. S2). Deoxyrhapontin elicited only a weak enhancement of CD41⁺CD42b⁺ cells at higher doses (Fig. S2), whereas emodin displayed a distinct and robust pro-megakaryocytic effect (Fig. 6), emerging as the only compound capable of consistently promoting MK-related phenotypes.

Given this clear functional superiority, subsequent studies focused on characterizing emodin’s thrombopoietic potential in depth. CCK-8 assays showed that emodin (2.5, 5, and 10 μM) did not affect proliferation at 1–3 days and only marginally suppressed growth at 5 days at the highest dose (Fig. S3A, C). Safety evaluation demonstrated that emodin (2.5, 5, and 10 μM) did not induce LDH release in either Meg-01 or K562 cells (Fig. S3B, D). Moreover, Emodin reduced basal apoptosis in both cell lines (Fig. S3E, F), establishing a safe and biologically favorable concentration range for differentiation studies. Using this range, we observed that emodin dose-dependently induced the appearance of large MK-like cells in Meg-01 and K562 cultures (Fig. 6A). Giemsa staining revealed prominent nuclear lobulation (Fig. 6B), and phalloidin staining confirmed multinucleation accompanied by cytoskeletal remodeling (Fig. 6C), all of which are canonical morphological hallmarks of MK differentiation. Consistent with these observations, emodin enhanced proportion of CD41⁺CD42b⁺ cells (Fig. 6D, F, H). DNA ploidy analysis further demonstrated that emodin promoted endomitosis-the defining process of MK maturation-characterized by a reduction of 2N cells and a corresponding expansion of the 4N–8N polyploid fractions (Fig. 6E, G, I). To confirm these findings in a more physiological context, emodin was next evaluated in primary murine HSPCs. After 7 days of treatment, HSPCs developed into large MK-like cells (Fig. 6J), and giemsa staining revealed typical multinuclear structures (Fig. 6K). Immunofluorescence for F-actin and β-tubulin further verified enhanced nuclear polyploidization and cytoskeletal rearrangement (Fig. 6L). Flow cytometric profiling demonstrated that emodin markedly increased both CD41⁺CD61⁺PI⁺ mature MKs and CD41⁺CD61⁺PI⁻ platelets in a dose-dependent manner (Fig. 6M–Q), establishing that emodin not only drives MK differentiation but also supports platelet biogenesis from primary progenitors. Together, these comprehensive cellular and primary progenitor assays firmly identify emodin as the dominant thrombopoietic component of RR, capable of promoting the full spectrum of MK development—from lineage commitment and endomitosis to terminal maturation and platelet production.

### Emodin accelerates platelet regeneration and mitigates radiation-induced bone marrow injury

**Fig. 7:**
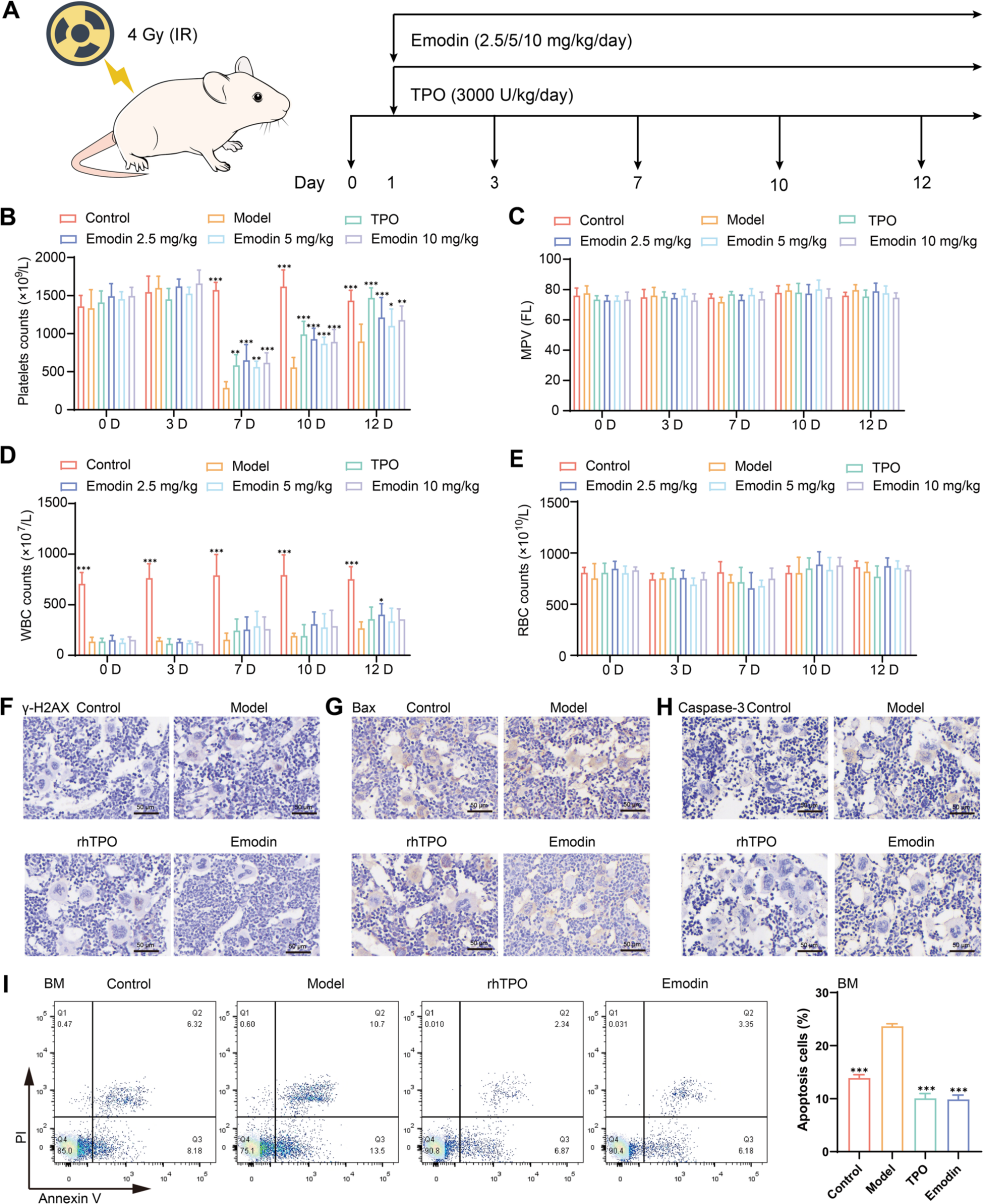
Emodin promotes platelet recovery and protects BM integrity in the RIT mouse. **A** RIT mouse model and treatment. Mice received 4 Gy whole-body X-ray irradiation followed by daily intraperitoneal administration of emodin (2.5, 5, or 10 mg/kg) or rhTPO (3000 U/kg) for 12 days. **B**–**E** Platelet counts **B**, MPV **C**, WBC **D** and **E** RBC counts measured on days 0, 3, 7, 10, and 12 after irradiation (n = 8). **F**–**H** Immunohistochemical staining of γ-H2AX **F**, Bax **G**, and Caspase-3 **H** of BM sections. Scale bars = 50 μm. **I** Apoptosis in BM cells detected by Annexin V/PI staining (n = 3). Data are presented as mean ± SD. For **B**–**E**, statistical significance was determined using two-way ANOVA; for I, one-way ANOVA was used. *p < 0.05, **p < 0.01, ***p < 0.001 vs. model group

Having verified emodin as the principal megakaryopoiesis-promoting constituent of RR in vitro, we next assessed its therapeutic efficacy and systemic safety in vivo using a RIT mouse model (Fig. 7A). In irradiated mice, platelet counts declined and reached their nadir on Day 7, followed by a gradual spontaneous recovery. Emodin (2.5, 5, and 10 mg/kg) treatment significantly attenuated this early decline and accelerated platelet rebound, yielding markedly higher platelet levels on days 10 and 12 (Fig. 7B). The absence of changes in MPV across all groups (Fig. 7C) suggests that the enhanced platelet output reflects increased production rather than alterations in platelet morphology. Emodin exerted only a mild, non-significant improvement in WBC recovery (Fig. 7D) and had no measurable effect on RBC counts (Fig. 7E), supporting its selective action on the megakaryocytic lineage under RIT conditions.

In parallel, a comprehensive safety evaluation of emodin was conducted under RIT conditions. Body weight monitoring showed that irradiation induced a marked loss of body weight, whereas emodin treatment significantly improved weight recovery during the observation period (Fig. S4A), indicating an overall amelioration of radiation-induced systemic injury. Importantly, emodin administration did not alter the relative organ indices of the heart, liver, spleen, lung, kidney, or thymus compared with the model or TPO-treated groups (Fig. S4B–G), suggesting the absence of overt organ hypertrophy or atrophy. Histopathological examination by H&E staining further confirmed that no pathological abnormalities or structural damage were observed in the heart, liver, lung, kidney, or thymus across all groups (Fig. S4H). Consistently, serum biochemical analyses revealed no significant differences in ALT, AST, AST/ALT ratio, UREA, LDH, or CK levels among groups (Fig. S4I–N), indicating preserved hepatic, renal, and systemic tissue integrity. Collectively, these data demonstrate that emodin is well tolerated in irradiated mice and exerts its thrombopoietic effects without inducing detectable systemic or organ-specific toxicity at therapeutically effective doses.

To investigate whether emodin also mitigates radiation-induced BM injury, we examined markers of DNA damage and apoptosis. Irradiation markedly increased γ-H2AX expression in BM sections, indicative of extensive DNA double-strand breaks; this signal was substantially reduced in emodin-treated mice (Fig. 7F). Likewise, the irradiation-induced upregulation of the pro-apoptotic proteins Bax and Caspase-3 was significantly suppressed following emodin administration (Fig. 7G, H). Flow cytometric analysis further confirmed that emodin effectively lowered the proportion of apoptotic BM cells induced by irradiation (Fig. 7I). Collectively, these data demonstrate that emodin exerts a dual protective function in vivo: it safely and selectively enhances thrombopoiesis by accelerating platelet regeneration, and simultaneously preserves the BM microenvironment by reducing DNA damage and limiting apoptotic loss of hematopoietic cells.

### Emodin drives multi-organ MK development and terminal maturation in RIT Mice

**Fig. 8:**
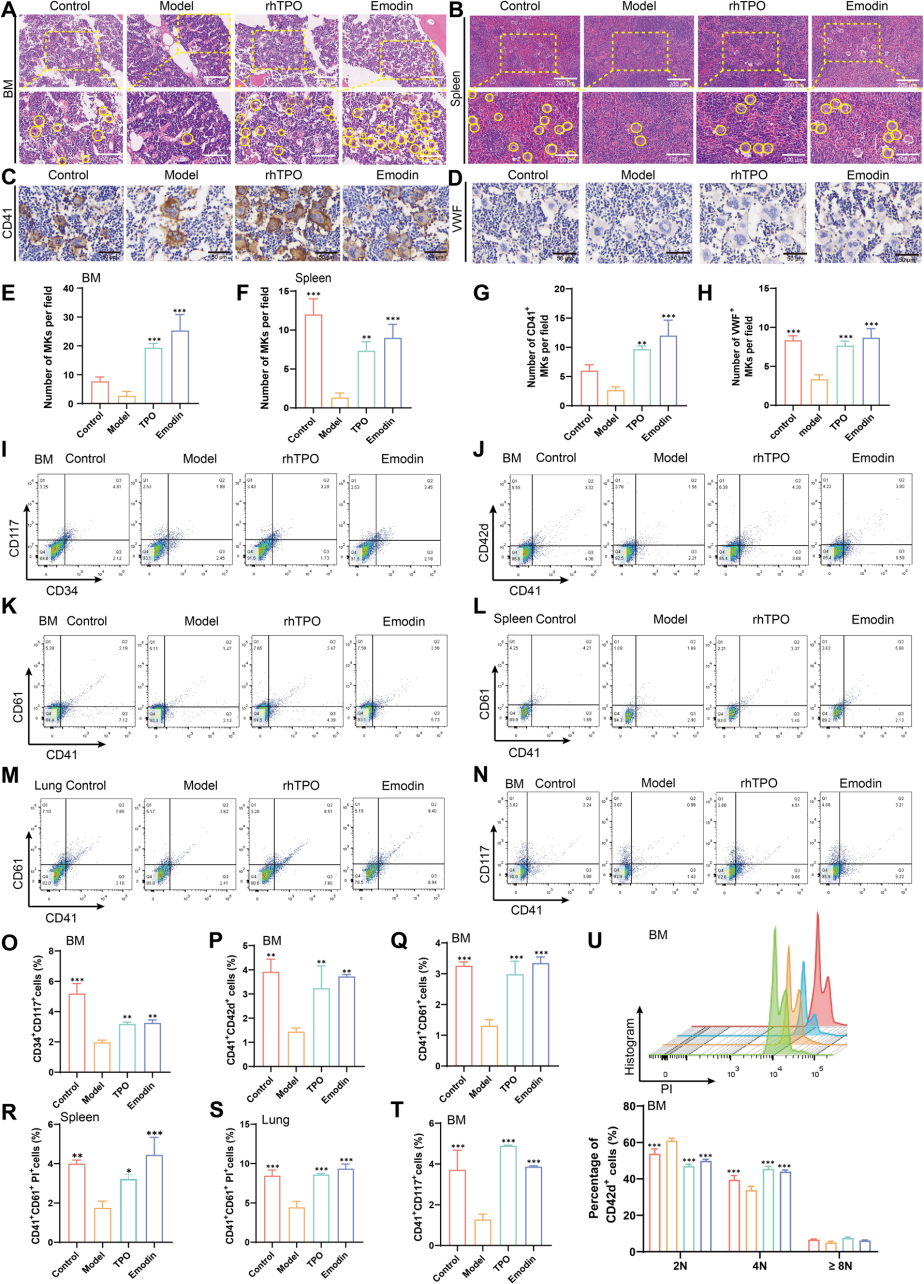
Emodin enhances megakaryocyte biogenesis, lineage commitment, and polyploid maturation in hematopoietic organs of RIT mice. **A**–**B** H&E staining of BM **A** and spleen **B** sections from Control, Model, rhTPO, and emodin-treated mice. Representative low- and high-magnification images are shown. MKs are indicated by yellow circles. Scale bars = 200 μm (upper panels), 100 μm (lower panels). **C**–**D** Immunohistochemical staining of BM sections for CD41 **C** and VWF **D**. Scale bars = 50 μm. **E**–**H** Quantification of MKs per field in BM **E**, spleen **F**, CD41⁺ MKs **G**, and VWF⁺ MKs **H** (n = 3). **I**–**N** Flow cytometric analyses of megakaryocytic lineage populations in BM, spleen, and lung: CD34⁺CD117⁺ early progenitors in BM **I**, CD117⁺CD41⁺ megakaryocytic progenitors in BM **J**, CD41⁺CD42d⁺ maturing MKs in BM **K**, CD41⁺CD61⁺ mature MKs in BM **L**, CD41⁺CD61⁺PI⁺ MKs in spleen **M**, and CD41⁺CD61⁺PI⁺ MKs in lung **N** (n = 3). **O** DNA ploidy distribution of bone marrow CD42d⁺ megakaryocytes analyzed by PI staining (n = 3). **P**–**U** Quantitative analyses corresponding to CD34⁺CD117⁺ cells in bone marrow **P**, CD117⁺CD41⁺ cells in bone marrow **Q**, CD41⁺CD42d⁺ cells in bone marrow **R**, CD41⁺CD61⁺ cells in bone marrow **S**, CD41⁺CD61⁺PI⁺ MKs in spleen **T**, and CD41⁺CD61⁺PI⁺ MKs in lung **U** (n = 3). Data are presented as mean ± SD. Statistical significance was determined using one-way ANOVA; for U, two-way ANOVA was used. *p < 0.05, **p < 0.01, ***p < 0.001 vs. Model group

To determine whether emodin-induced platelet recovery reflected enhanced MK production, we analyzed BM, spleen, and lung tissues. Histological, immunohistochemical, and multiparametric flow cytometric assays were performed to assess MK development and platelet-producing capacity in these hematopoietic compartments [26, 30]. H&E staining revealed that irradiation caused a marked depletion of MKs in both BM and spleen (Fig. 8A, B, E, F). Treatment with emodin (10 mg/kg) or rhTPO markedly increased MK abundance, with conspicuous clusters of large, mature MKs readily observed in both tissues (Fig. 8A, B). Quantification confirmed a robust elevation in MK number relative to model group (Fig. 8A, B, E, F), indicating that emodin effectively restores de novo MK formation in both medullary and extramedullary hematopoietic niches. The restoration of MK lineage output was further substantiated by immunohistochemical staining for CD41 and VWF. Irradiation sharply reduced CD41⁺ and VWF⁺ MKs, whereas emodin and rhTPO treatment substantially increased both markers (Fig. 8C, D, G, H), demonstrating that emodin not only replenishes MK cellularity but also promotes their progression toward mature, platelet-producing phenotypes. To delineate the hierarchy of hematopoietic recovery, we next profiled MK development using flow cytometry. Emodin significantly increased early progenitor populations (CD34⁺CD117⁺) (Fig. 8I, O) and megakaryocytic-committed progenitors (CD117⁺CD41⁺) (Fig. 8J, P) in the BM, indicating enhanced lineage priming and progenitor expansion. Emodin also elevated the proportions of more differentiated MK subsets, including CD41⁺CD42d⁺ (Fig. 8K, Q) and CD41⁺CD61⁺ cells (Fig. 8L, R), confirming its ability to drive intermediate and late MK maturation. Emodin also promoted MK maturation beyond the BM. In the spleen—a key site of stress-induced extramedullary megakaryopoiesis—emodin increased CD41⁺CD61⁺PI⁺ mature MKs (Fig. 8M, S). A similar enhancement was observed in the lung (Fig. 8N, T), where terminal MK fragmentation and platelet release predominantly occur, suggesting that emodin supports a coordinated, multi-organ program of platelet biogenesis. Finally, we assessed the terminal maturation checkpoint of polyploidization. DNA ploidy analysis of CD42d⁺ BM MKs demonstrated that emodin significantly reduced the proportion of immature 2N MKs while increasing the proportion of 4N cells (Fig. 8U). This shift toward high-ploidy MKs is a defining prerequisite for efficient platelet production and provides direct evidence that Emodin accelerates endomitosis during MK maturation. Taken together, these findings establish that emodin exerts a comprehensive megakaryopoietic effect in vivo—expanding progenitor pools, enhancing lineage commitment, restoring MK differentiation, and promoting terminal polyploidization across BM, spleen, and lung. This coordinated enhancement of the entire MK developmental continuum provides a mechanistic basis for the potent thrombopoietic activity observed in irradiated mice.

### Network pharmacology and transcriptomic profiling define key signaling axes engaged by emodin during megakaryopoiesis

**Fig. 9:**
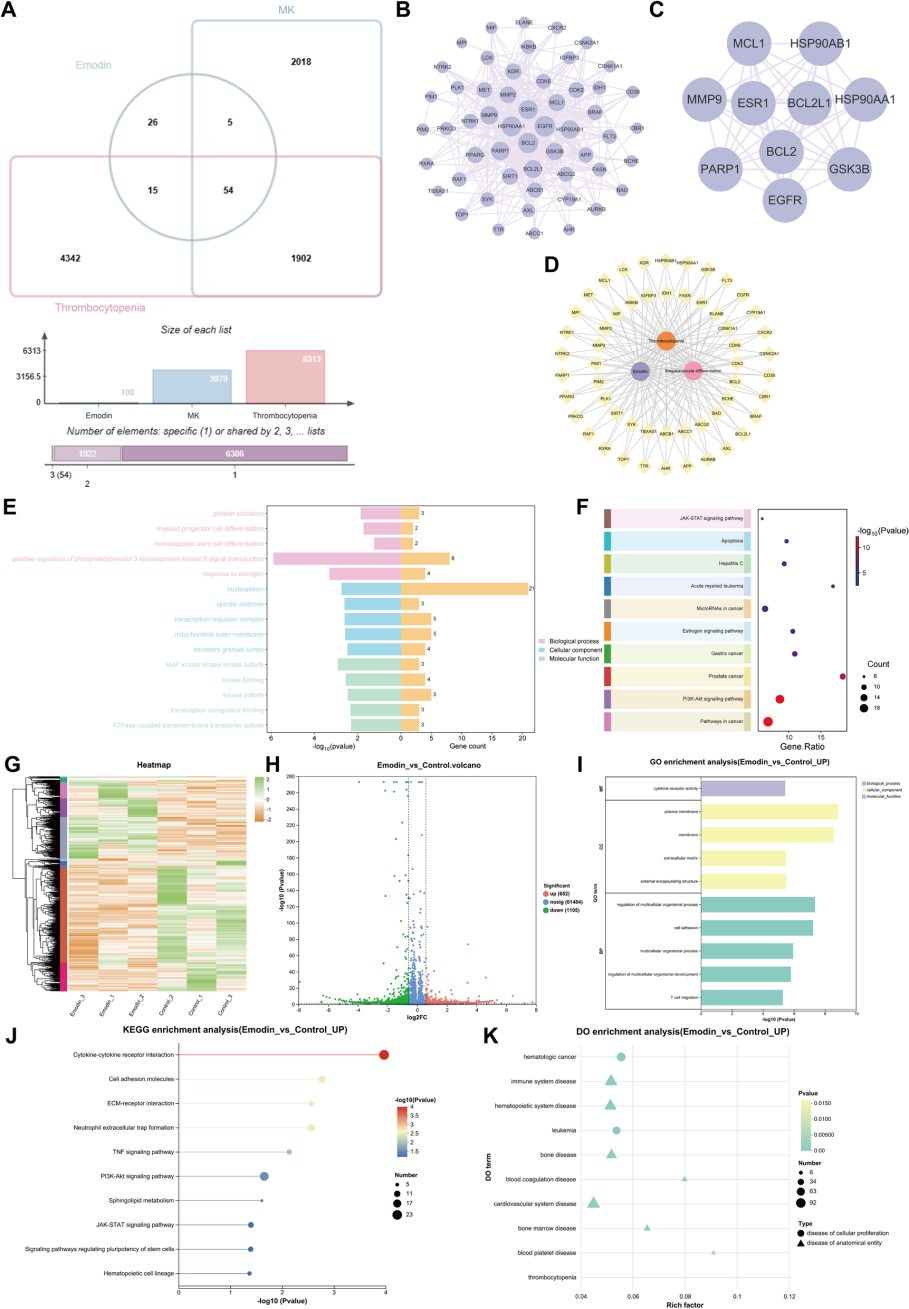
Network pharmacology prediction and transcriptomic profiling identify putative emodin-responsive pathways in megakaryocyte biology. **A** Venn diagram of emodin-predicted targets and genes associated with MK differentiation and thrombocytopenia. **B** PPI network of overlapping emodin–MK differentiation– thrombocytopenia related targets constructed using STRING and visualized in Cytoscape. **C** Subnetwork showing core regulatory nodes (degree > 10) derived from PPI analyses. **D** Integrated emodin–target–MK differentiation–thrombocytopenia interaction network illustrating compound–target–disease associations. **E** GO enrichment of targets identified by network pharmacology. **F** KEGG pathway enrichment of overlapping targets identified by network pharmacology. **G** Heatmap showing hierarchical clustering of DEGs in Meg-01 cells treated with emodin (10 μM) versus control. **H** Volcano plot of DEGs between emodin-treated and control Meg-01 cells. **I** GO enrichment analysis of upregulated DEGs categorized into BP, CC, and MF domains. **J** KEGG enrichment of upregulated DEGs. **K** DO enrichment analysis of upregulated DEGs

To elucidate the molecular mechanisms underlying emodin’s thrombopoietic activity, we first performed a systematic network pharmacology analysis. Potential emodin targets predicted by SwissTargetPrediction and TCMSP were standardized and merged, then intersected with genes associated with MK differentiation and thrombocytopenia collected from GeneCards, OMIM, and TTD. Venn analysis identified a substantial set of shared targets between emodin and both MK differentiation and thrombocytopenia (Fig. 9A). Construction of PPI networks revealed a densely connected regulatory architecture (Fig. 9B–C), within which ESR1 emerged as one of the highest-degree hub nodes (Fig. 9C). Integration of emodin–target–MK differentiation–thrombocytopenia interactions (Fig. 9D) demonstrated that emodin is predicted to engage multiple signaling axes implicated in hematopoietic regulation. GO enrichment highlighted biological processes related to platelet activation, myeloid progenitor cell differentiation, hematopoietic stem cell differentiation, and response to estrogen; cellular components involving nucleoplasm and spindle midzone; and molecular functions enriched for MAP kinase kinase kinase activity, and kinase binding (Fig. 9E). KEGG analysis further implicated estrogen, PI3K–AKT, and JAK–STAT pathways (Fig. 9F), implying that estrogen-related signaling—including ESR1—and the associated PI3K–AKT and JAK–STAT pathways may be among the pathways potentially engaged by emodin in regulating megakaryocyte biology.

To validate these predicted mechanisms at the transcriptional level, we conducted RNA sequencing of Meg-01 cells treated with emodin for 3 days. Clustering revealed a clear segregation between control and treated samples, indicating that emodin induces a reproducible transcriptional program (Fig. 9G). Differential expression analysis identified 652 up-regulated and 1105 down-regulated genes (Fig. 9H). GO enrichment of the up-regulated genes revealed significant activation of cytokine receptor activity (MF), extracellular matrix and plasma membran (CC), and BP such as regulation of multicellular organismal process, and cell adhesion (Fig. 9I). These enriched terms reflect enhanced responsiveness to cytokine cues, strengthened cell–matrix dynamics, and activation of developmental programs consistent with MK lineage progression. KEGG analysis further demonstrated enrichment in “Cytokine–cytokine receptor interaction,” “Hematopoietic cell lineage,” “PI3K–Akt signaling pathway,” “JAK–STAT signaling pathway,” and adhesion-associated pathways such as “ECM–receptor interaction” and “Cell adhesion molecules” (Fig. 9J), aligning well with the network pharmacology predictions. Disease Ontology (DO) enrichment linked the emodin-upregulated gene signature to hematopoietic system disorders, blood coagulation and platelet diseases, bone marrow disease, and thrombocytopenia (Fig. 9K), underscoring its relevance to hematopoietic recovery. Taken together, the integration of network pharmacology and transcriptomic profiling demonstrates that emodin engages a coherent regulatory network centered on estrogen signaling, hematopoietic lineage determination, and hematopoietic lineage differentiation pathways such as PI3K–AKT and JAK–STAT. These findings demonstrate that emodin activates the molecular circuitry required for MK differentiation, endomitosis, and platelet production, supporting its therapeutic potential in thrombocytopenia.

### Emodin directly targets ESR1 to drive MK differentiation

**Fig. 10:**
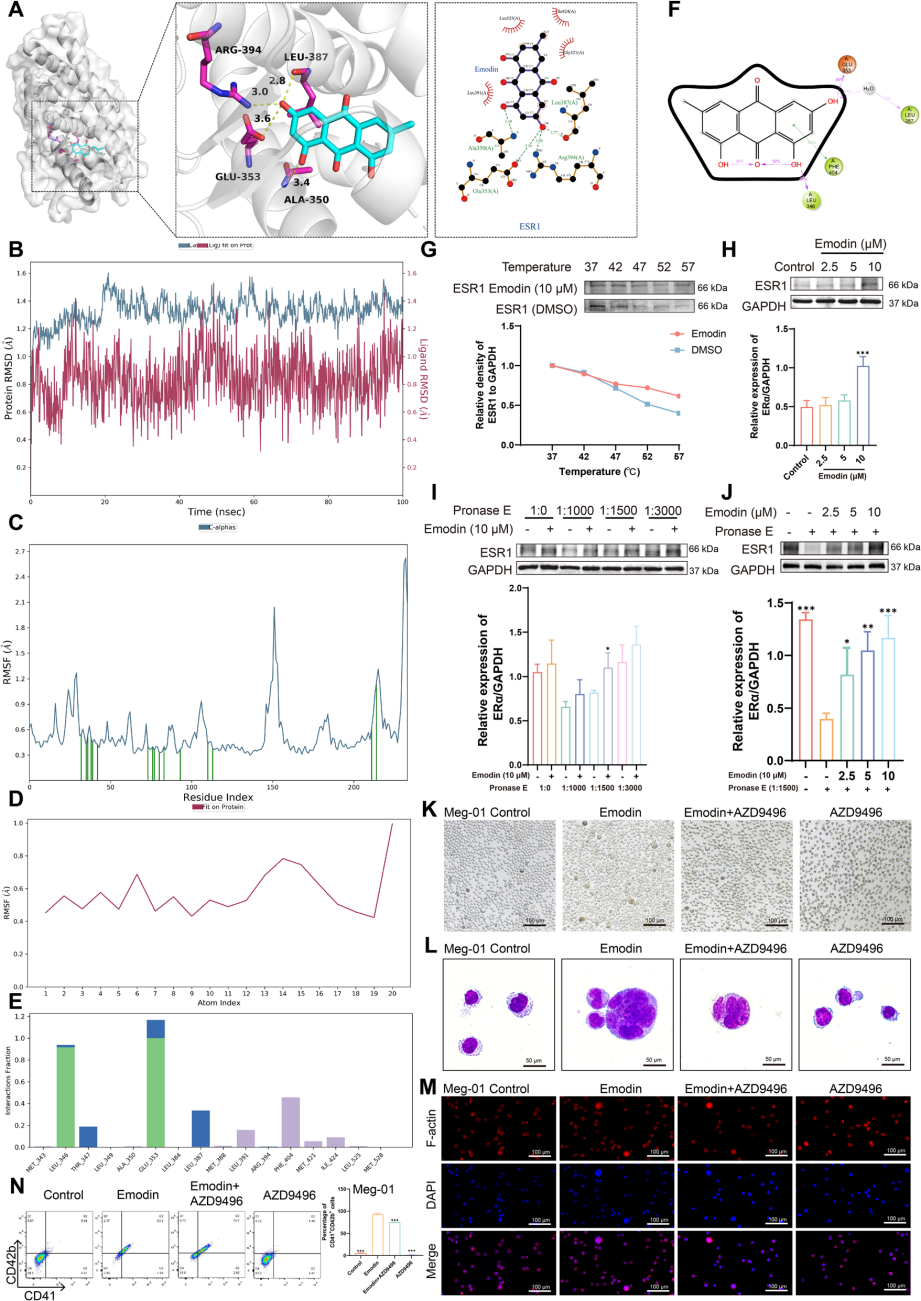
Emodin directly binds to ESR1 and functionally requires ESR1 to promote MK differentiation. **A** Molecular docking model showing the predicted binding pose of emodin within ligand-binding domain of ESR1. **B** Molecular dynamics simulation of the ESR1–emodin complex showing RMSD trajectories for protein backbone and ligand over the simulation period. **C**–**D** RMSF analyses of ESR1 residues **C** and emodin **D** heavy atoms during molecular dynamics simulation, indicating residue-level flexibility and ligand stability. **E**–**F** Interaction fraction diagrams summarizing hydrogen bonds, hydrophobic contacts, water bridges, and intramolecular hydrogen bonds formed between ESR1 and emodin. **G** CETSA evaluating ESR1 thermal stability in Meg-01 lysates incubated with emodin (2.5, 5, 10 μM) or vehicle and heated at 37–57 °C for 10 min (n = 3). **H** Quantification of ESR1 thermal stabilization at 52 °C under increasing concentrations of emodin (n = 3). **I** DARTS assay showing protease protection of ESR1 in the presence of emodin across different Pronase E dilutions (1:1000, 1:1500, 1:3000) (n = 3). **J** Concentration-dependent protection of ESR1 from Pronase E digestion (1:1500 condition) by emodin (n = 3). **K** Representative bright-field images of Meg-01 cells treated with emodin (10 μM) with or without the ESR1 inhibitor AZD9496 (3 μM). Scale bars = 100 μm. **L** Giemsa staining showing nuclear morphology under the same treatment conditions. Scale bars = 50 μm. **M** Phalloidin staining demonstrating changes in nuclear number and cytoskeletal architecture. Scale bars = 100 μm. **N** CD41⁺CD42b⁺ MK populations following treatment with emodin alone or in combination with AZD9496 (n = 3). Data are presented as mean ± SD. Statistical significance was determined using one-way ANOVA; for G, two-way ANOVA was used. *p < 0.05, **p < 0.01, ***p < 0.001 vs. control group

Given that network pharmacology and transcriptomic analyses repeatedly highlighted ESR1 as a putative regulatory node for emodin, we next investigated whether ESR1 functions as a direct molecular target mediating emodin-induced MK differentiation. Molecular docking predicted a stable binding interface between emodin and ESR1, with the ligand occupying a well-defined pocket that engages multiple residues through hydrogen bonding and hydrophobic interactions (Fig. 10A). Notably, LEU387, ARG394, and ALA350 formed direct hydrogen bonds with emodin, whereas GLU353 exhibited a hydrogen-bonding occupancy of 99% during molecular dynamics simulation (Fig. 10E–F), indicating its critical role in ligand stabilization. The calculated binding energy of –7.8 kcal/mol further supported a favorable interaction. Molecular dynamics simulations demonstrated minimal RMSD fluctuations for both ESR1 and emodin (protein ~ 1.4 Å; ligand ~ 0.4–1.4 Å), suggesting robust conformational stability throughout the simulation (Fig. 10B). Residue-level RMSF analysis confirmed that the interacting residues displayed low flexibility (Fig. 10C–D), consistent with stable ligand engagement. The predicted emodin-binding site is located within the canonical ligand-binding pocket of the ESR1 ligand-binding domain rather than at a distant allosteric site. However, the interaction pattern of emodin differs from that of endogenous estradiol, involving a partially distinct network of hydrogen-bonding and hydrophobic contacts with residues such as GLU353, LEU387, ARG394, and ALA350. These structural features suggest that emodin may act as a non-steroidal ESR1 ligand/modulator that engages the canonical pocket through a non-classical binding mode. To experimentally validate the predicted ESR1–emodin interaction, we performed CETSA. ESR1 in emodin-treated Meg-01 lysates exhibited markedly increased thermal stability compared with controls across a temperature range of 37–57 °C (Fig. 10G, Fig. S5). At 52 °C, ESR1 stabilization was dose dependent, with 10 μM emodin producing the strongest protection (Fig. 10H, Fig. S5). Consistent findings were obtained using DARTS, where ESR1 showed enhanced resistance to proteolytic degradation by Pronase E in the presence of emodin (Fig. 10I, Fig. S5). Furthermore, emodin dose-dependently protected ESR1 from digestion at a fixed Pronase E concentration (1:1500), reinforcing the conclusion that emodin physically binds ESR1 and stabilizes its structure (Fig. 10J, Fig. S5).

**Fig. 11:**
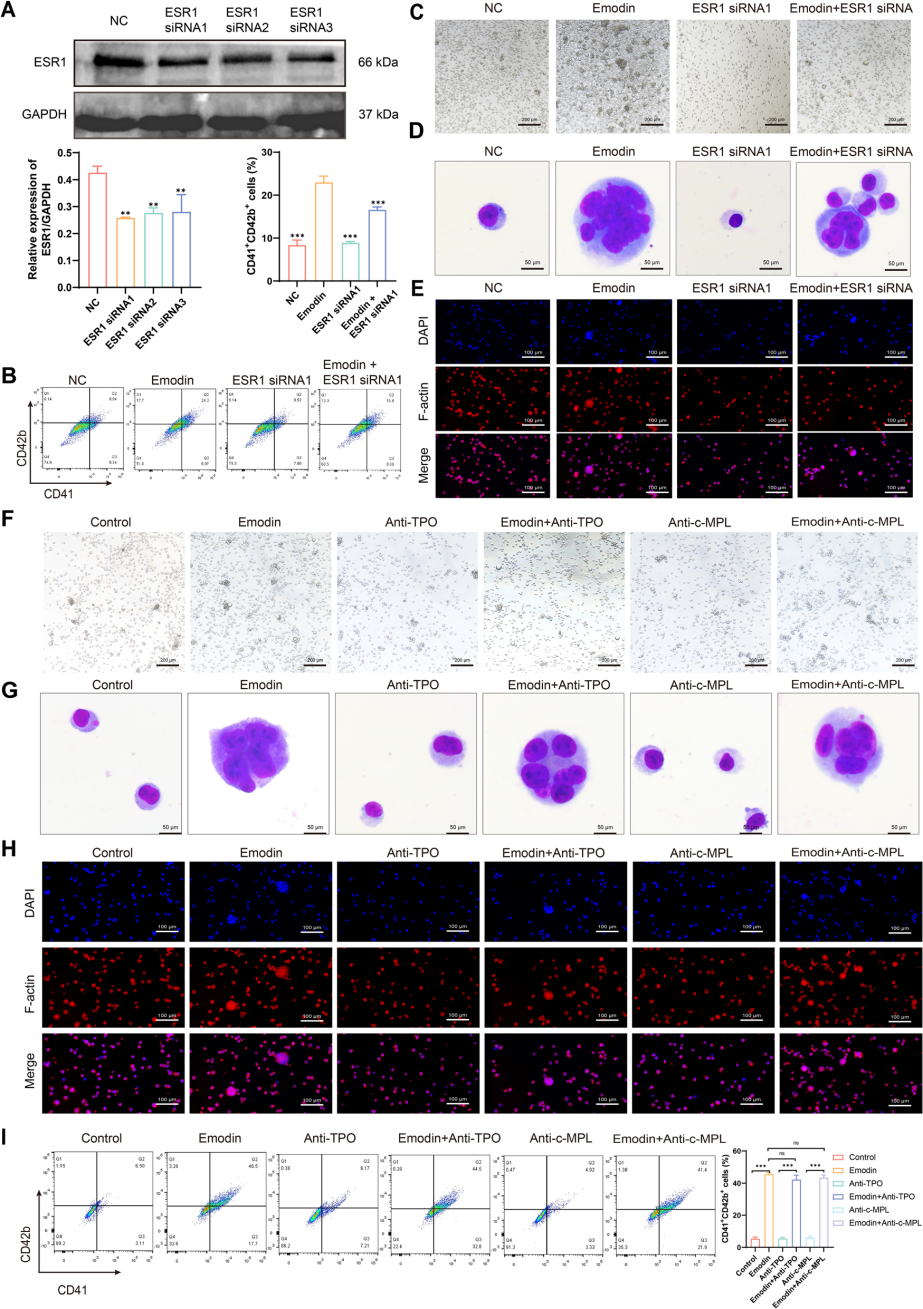
ESR1 knockdown attenuates emodin-induced MK differentiation, and TPO/c-MPL neutralization does not abolish the pro-megakaryocytic effect of emodin. **A** Western blot analysis of ESR1 knockdown efficiency in Meg-01 cells transfected with NC, ESR1 siRNA1, ESR1 siRNA2, or ESR1 siRNA3. Quantification of ESR1 protein expression is shown below (n = 3). **B** Flow cytometric analysis and quantification of CD41⁺CD42b⁺ cells in NC, emodin, ESR1 siRNA1, and emodin + ESR1 siRNA1 groups (n = 3). **C** Representative bright-field images showing MK-like morphological changes after ESR1 knockdown and/or emodin treatment. Scale bars = 200 μm. **D** Giemsa staining showing nuclear enlargement and lobulation. Scale bars = 50 μm. **E** Immunofluorescence staining of DAPI and F-actin showing multinucleation and cytoskeletal remodeling. Scale bars = 100 μm. **F** Representative bright-field images of Meg-01 cells treated with emodin in the presence or absence of anti-TPO or anti-c-MPL neutralizing antibodies. Scale bars = 200 μm. **G** Giemsa staining showing MK-like nuclear morphology after TPO or c-MPL neutralization. Scale bars = 50 μm. **H** Immunofluorescence staining of DAPI and F-actin showing nuclear morphology and cytoskeletal remodeling. Scale bars = 100 μm. **I** Flow cytometric analysis and quantification of CD41⁺CD42b⁺ cells after treatment with emodin, anti-TPO, emodin + anti-TPO, anti-c-MPL, or emodin + anti-c-MPL (n = 3). Data are presented as mean ± SD. Statistical significance was determined using one-way ANOVA. ns, not significant; **p < 0.01, ***p < 0.001 vs. the indicated groups

We then evaluated whether ESR1 is functionally required for emodin-induced MK differentiation. Exposure of Meg-01 cells to the ESR1 antagonist AZD9496 (3 μM) markedly attenuated the morphological and phenotypic features elicited by emodin (10 μM). Specifically, AZD9496 blocked the appearance of enlarged MK-like cells (Fig. 10K), prevented nuclear lobulation (Fig. 10L), reduced multinucleation events (Fig. 10M), and significantly suppressed the emodin-induced increase in CD41⁺CD42b⁺ MKs (Fig. 10N). These findings demonstrate that ESR1 activity is indispensable for the pro-differentiation effects of emodin. To further substantiate the requirement of ESR1 using a genetic approach, we performed ESR1 knockdown experiments in Meg-01 cells. Three independent ESR1-targeting siRNAs were first evaluated, and western blot analysis showed that ESR1 siRNA1 produced the most efficient reduction of ESR1 protein expression; therefore, ESR1 siRNA1 was selected for subsequent functional experiments (Fig. 11A). Consistent with the pharmacological inhibition results, ESR1 knockdown markedly weakened the pro-megakaryocytic effect of emodin. Flow cytometric analysis showed that emodin significantly increased the proportion of CD41⁺CD42b⁺ cells, whereas ESR1 siRNA1 substantially attenuated this increase (Fig. 11B). Morphological observation further demonstrated that ESR1 silencing reduced emodin-induced formation of enlarged MK-like cells (Fig. 11C). Giemsa staining showed that ESR1 knockdown impaired emodin-induced nuclear enlargement and lobulation (Fig. 11D). In parallel, F-actin staining revealed that ESR1 siRNA1 attenuated the multinucleation and cytoskeletal remodeling induced by emodin (Fig. 11E). These results provide genetic evidence that ESR1 is required for emodin-induced MK differentiation and further support ESR1 as a functional molecular target of emodin.

Because TPO/c-MPL represents the classical ligand–receptor system governing MK development, we next examined whether emodin-induced MK differentiation required this pathway. To directly address this issue, Meg-01 cells were treated with emodin in the presence or absence of neutralizing antibodies against TPO or c-MPL. Emodin markedly induced enlarged MK-like morphology, nuclear lobulation, multinucleation, F-actin remodeling, and expansion of the CD41⁺CD42b⁺ population (Fig. 11F–I). Importantly, neither anti-TPO nor anti-c-MPL antibody treatment abolished the pro-differentiation effects of emodin; emodin still maintained evident MK-like morphological changes and a high proportion of CD41⁺CD42b⁺ cells under TPO or c-MPL blockade (Fig. 11F–I). These results indicate that emodin-induced MK differentiation does not require endogenous TPO or c-MPL activation, thereby supporting ESR1, rather than the classical TPO/c-MPL system, as the functionally relevant target mediating the pro-megakaryocytic activity of emodin. Collectively, computational prediction, molecular dynamics simulation, CETSA/DARTS-based target engagement assays, pharmacological ESR1 blockade, ESR1 siRNA1-mediated knockdown, and TPO/c-MPL neutralization experiments converge to establish ESR1 as a direct and functionally required molecular target through which emodin promotes megakaryocytic lineage progression, while excluding an essential dependence on the classical TPO/c-MPL system.

### Activation of PI3K/AKT and JAK2/STAT3 pathways defines the downstream mechanism of ESR1-mediated emodin activity

**Fig. 12:**
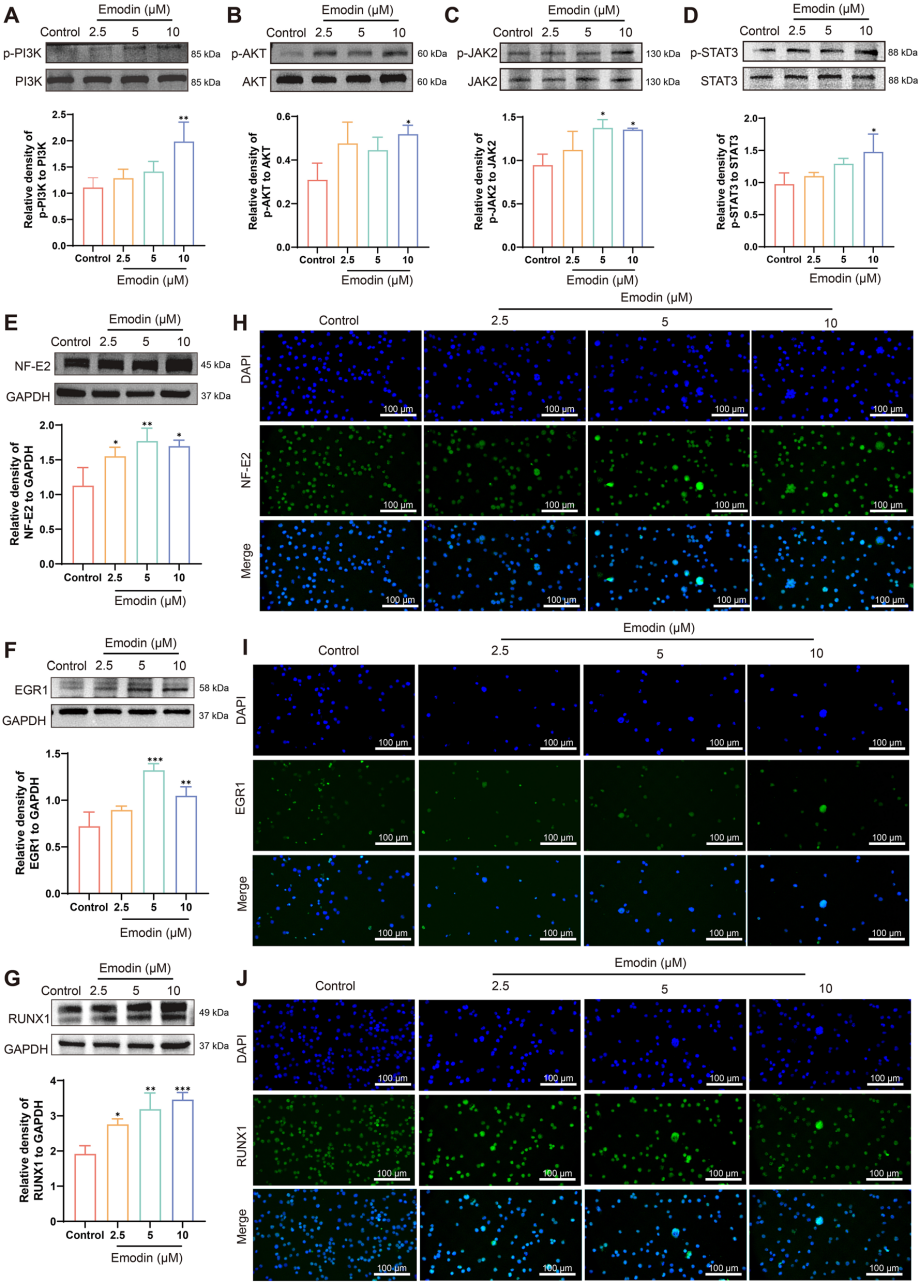
Emodin activates PI3K/AKT and JAK2/STAT3 signaling. **A**–**D** Western blot analyses of phosphorylated PI3K **A**, AKT **B**, JAK **C**, and STAT3 **D** in Meg-01 cells treated with emodin, with corresponding quantitative densitometry shown on the right (n = 3). **E**–**G** Western blot detection of NF-E2 **E**, EGR1 **F**, and RUNX1 **G** following emodin treatment, with quantification of band intensities (n = 3). **H**–**J** Immunofluorescence staining of NF-E2 **H**, EGR1 **I**, and RUNX1 **J** in Meg-01 cells. Scale bar = 100 μm. Data are presented as mean ± SD. Statistical significance was determined using one-way ANOVA. *p < 0.05, **p < 0.01, ***p < 0.001 vs. control group

**Fig. 13:**
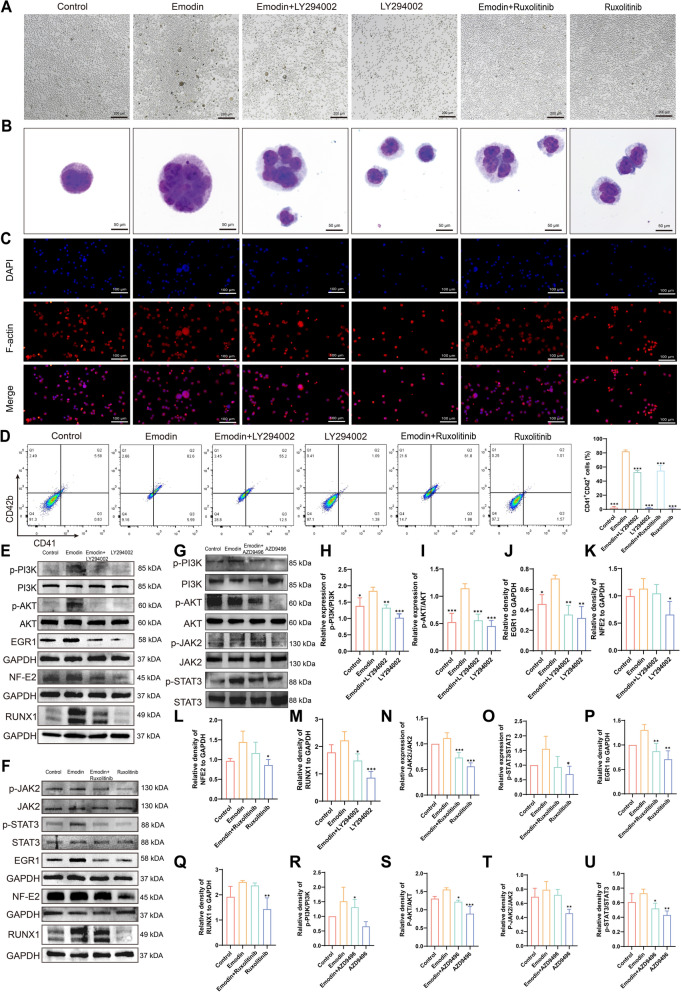
Inhibition of PI3K/AKT or JAK2/STAT3 signaling abrogates emodin-induced MK differentiation. **A** Meg-01 cells under the indicated treatments: Control, Emodin, Emodin + LY294002, LY294002, Emodin + Ruxolitinib, and Ruxolitinib. Scale bar = 200 μm. **B** Giemsa staining of Meg-01 cells showing nuclear morphology and MK-like cellular enlargement across treatment groups. Scale bar = 50 μm. **C** Immunofluorescence imaging of F-actin (red) and nuclei (DAPI, blue) illustrating cytoskeletal remodeling and multinucleation under different treatments. Scale bar = 100 μm. **D** CD41⁺CD42b⁺ MK populations in each treatment group, with quantitative summary at right (n = 3). **E**–**U** Western blot analyses of p-PI3K, PI3K, p-AKT, AKT, EGR1, NF-E2, and RUNX1 in Meg-01 cells treated with emodin in the presence or absence of LY294002, ruxolitinib, or AZD9496 with densitometric quantification corresponding to each protein (n = 3). Data are presented as mean ± SD. Statistical significance was determined using one-way ANOVA. *p < 0.05, **p < 0.01, ***p < 0.001 vs. emodin group

Having established ESR1 as a direct and functionally relevant molecular target of emodin, we next investigated whether downstream signaling pathways predicted by network pharmacology and transcriptome analyses were engaged during emodin-induced MK differentiation. Both analyses consistently highlighted ESR1-associated signaling modules-particularly PI3K/AKT and JAK2/STAT3-as major regulatory pathways linked to megakaryopoiesis. These pathways are well recognized as critical mediators of MK endomitosis and terminal maturation, suggesting that ESR1 activation might converge on these canonical hematopoietic signaling cascades. Consistent with this mechanistic framework, western blot analyses demonstrated that emodin treatment enhanced the phosphorylation levels of PI3K and AKT (Fig. 12A–B, Fig. S5), and phosphorylated JAK2 and STAT3 (Fig. 12C–D, Fig. S5). These changes occurred without major alterations in the corresponding total protein bands, indicating activation rather than upregulation of these pathways. Given the essential role of lineage-determining transcription factors in MK differentiation and maturation, we further assessed NF-E2, EGR1, and RUNX1. Emodin increased the expression of all three transcription factors (Fig. 12E–G, Fig. S5), and immunofluorescence confirmed their enhanced nuclear localization (Fig. 12H–J), consistent with activation of a differentiation program downstream of PI3K/AKT and JAK2/STAT3. To determine whether activation of these signaling pathways was required for emodin-induced differentiation, we pharmacologically inhibited PI3K/AKT or JAK2/STAT3 signaling using LY294002 (10 μM) and ruxolitinib (0.5 μM), respectively. Blockade of either pathway markedly attenuated the characteristic features of emodin-induced MK maturation, including the formation of enlarged MK-like cells (Fig. 13A), nuclear lobulation (Fig. 13B), increased nuclear number (Fig. 13C), and the rise in CD41⁺CD42b⁺ populations (Fig. 13D). Furthermore, both inhibitors substantially suppressed emodin-induced phosphorylation of PI3K/AKT or JAK2/STAT3, respectively, and diminished NF-E2, EGR1, and RUNX1 expressions (Fig. 13E–P, Fig. S6), confirming that these signaling cascades are indispensable for the differentiation program driven by emodin. Importantly, pharmacological blockade of ESR1 using the selective antagonist AZD9496 markedly attenuated emodin-induced phosphorylation of PI3K, AKT, JAK2, and STAT3 (Fig. 13G, R–U, Fig. S7), providing direct biochemical evidence that activation of both PI3K/AKT and JAK2/STAT3 signaling is ESR1-dependent. Taken together, the convergence of evidence from target prediction, transcriptomic profiling, molecular docking and dynamics, biochemical target engagement assays (CETSA/DARTS), and pathway inhibition experiments supports a coherent mechanistic model in which emodin activates ESR1 and subsequently engages the PI3K/AKT and JAK2/STAT3 signaling cascades to drive megakaryocytic lineage progression.

## Discussion

Ionizing radiation is indispensable in modern oncology but its myelotoxicity remains a major limitation [1]. Among hematologic toxicities, RIT is particularly problematic because platelets have a short life span and limited storage options, and severe thrombocytopenia exposes patients to life-threatening bleeding [31]. Current interventions—platelet transfusion, rhTPO and thrombopoietin receptor agonists—are constrained by short supply, cost, immunogenicity, thrombotic risk, and the fact that they largely act on residual MKs without repairing global bone marrow damage [32–34]. Thus, agents that both protect the hematopoietic microenvironment and actively drive megakaryopoiesis are highly desirable as adjuncts to radiotherapy.

TCM and natural products offer a rich source of such candidates. Several groups, including ours, have shown that complex herbal preparations or purified phytochemicals can promote hematopoietic recovery after chemo- or radiotherapy, often through multi-target regulation of oxidative stress, inflammatory cytokines, and hematopoietic signaling networks [14–16, 35]. In this context, RR is intriguing. Classically used for purgation, “clearing heat,” and promoting blood circulation, RR has also been used empirically to manage hemorrhagic complications [19]. Here we provide systematic experimental evidence that RR exerts robust anti-RIT activity: it ameliorates radiation-induced DNA damage and apoptosis in BM, restores MK output in BM and spleen, promotes MK maturation and polyploidization, and ultimately accelerates platelet recovery. Network pharmacology and transcriptomic analyses indicate that RR does so via coordinated modulation of multiple signaling pathways rather than a single linear axis, which is consistent with the multi-component nature of this herb.

From a pharmacological standpoint, bridging complex herbal mixtures with defined molecular entities is essential for mechanism-based drug discovery. By screening eight representative RR constituents in MK-lineage cells, we identified emodin as the dominant in vitro driver of megakaryopoiesis. Deoxyrhapontin showed only weak activity, whereas the other components were essentially inactive under the conditions tested. This finding aligns with the pleiotropic pharmacology of emodin reported in other systems. Emodin, an anthraquinone derivative, exhibits anti-inflammatory, anti-tumor, metabolic, and neuroprotective properties [20–23]. In our previous work using a chronic constriction injury model, emodin reversed neuropathic pain behaviors and normalized spinal inflammatory cytokine profiles. It also re-programmed the transcriptome and metabolome of spinal cord tissue, partly through suppression of P2X4-p38 MAPK–NF-κB signaling in microglia [20]. These data underscore that emodin is not merely an antioxidant, but a bona fide signal-modulating small molecule. Nevertheless, the strong MK-promoting activity of emodin in vitro does not mean that other RR components are irrelevant in vivo. Their metabolites may be active, and “inactive” compounds may modulate pharmacokinetics, tissue distribution, local inflammation, or vascular responses. These effects could cooperatively enhance the overall radioprotective and thrombopoietic activity of RR. Therefore, RR should be discussed as an “emodin-centered but multi-component” herbal intervention, rather than being reduced to a single chemical entity.

The in vivo results demonstrate that emodin exerts dual effects by promoting thrombopoiesis and mitigating radiation-induced BM injury. In the RIT model, emodin selectively restored platelet counts without significantly affecting erythrocytes or leukocytes, and did so without altering mean platelet volume, indicating genuine stimulation of platelet production rather than a shift in platelet size. Importantly, emodin also reduced γ-H2AX accumulation and attenuated Bax/Caspase-3-mediated apoptosis in BM cells, thereby preserving the hematopoietic niche. This dual action contrasts with classical TPO mimetics, which typically act downstream on MK progenitors but do not directly mitigate DNA damage. Furthermore, emodin expanded HSPCs (CD34⁺CD117⁺), MK-committed progenitors (CD117⁺CD41⁺), and successive maturation stages (CD41⁺CD42d⁺, CD41⁺CD61⁺), and enhanced polyploidization of CD42d⁺ MKs. Emodin promoted megakaryopoiesis across the BM, spleen, and lung, with increased mature and platelet-producing MKs in all three tissues. This pattern suggests a coordinated reinforcement of thrombopoiesis, spanning progenitor expansion, lineage commitment, and terminal platelet shedding. This global reshaping of MK biology is reminiscent of other stress-thrombopoiesis paradigms driven by non-TPO signals, such as arginine vasopressin (AVP)-AKT–dependent proplatelet formation, CCL5–CCR5-mediated MK ploidy increase, or tyrosyltRNA synthetase (YRS^ACT^) -induced MK-biased hematopoiesis [36–38]. Emodin thus joins a growing list of molecules that can bypass or complement classical TPO/c-MPL signaling to rapidly restore platelets under stress. The safety profile of emodin should also be interpreted cautiously. As an anthraquinone derivative, emodin has been reported to cause genotoxicity, nephrotoxicity, or other adverse effects under high-dose or long-term exposure conditions [21, 22]. In the present study, emodin was administered at relatively low doses (2.5–10 mg/kg) for a short therapeutic period. Within this treatment window, emodin promoted platelet recovery and BM repair without obvious systemic toxicity, as reflected by improved body weight recovery, unchanged organ indices, absence of overt histopathological injury in major organs, and stable serum biochemical parameters including ALT, AST, AST/ALT ratio, UREA, LDH, and CK. These findings suggest a favorable short-term balance between efficacy and tolerability under the present RIT treatment schedule. However, they do not establish long-term safety. Future studies should evaluate chronic toxicity, genotoxicity, renal safety, pharmacokinetic/pharmacodynamic profiles, dose-exposure relationships, and the therapeutic window before emodin can be considered for repeated or long-term thrombocytopenia management.

Mechanistically, our network pharmacology and RNA sequencing analyses converged on ESR1 as a central target of emodin. Estrogen receptors have been implicated in thrombopoiesis, but most mechanistic work has focused on ERβ. An elegant study demonstrated that estrogen enhances MK maturation via ERβ-mediated activation of GATA1, which in turn upregulates STAT1 and NF-E2, ultimately driving thrombopoiesis [10]. In contrast, the role of ESR1 (ERα) in MK biology has been far less explored. Our data indicate that ESR1 is not only expressed in MK-lineage cells but also serves as a functionally relevant receptor for emodin. Docking and molecular dynamics simulations revealed that emodin binds within the canonical ligand-binding pocket of ESR1, engaging residues such as Glu353, Leu387, and Arg394 and forming a stable interaction network with favorable binding energy. CETSA and DARTS experiments in Meg-01 cells confirmed that emodin markedly stabilizes ESR1 against thermal and proteolytic denaturation, providing biochemical evidence of direct binding in a cellular context. Functionally, the ESR1 antagonist AZD9496 and ESR1 siRNA knockdown attenuated emodin-induced MK enlargement, nuclear lobulation, multinuclearity, and CD41⁺CD42b⁺ accumulation, demonstrating that ESR1 activity is required for emodin’s promegakaryocytic effects. Together, these findings suggest that emodin may function as a non-steroidal ESR1 modulator in MKs, representing a mechanistically distinct, estrogen receptor–dependent route toward thrombopoiesis. Structurally, emodin is predicted to occupy the canonical ESR1 ligand-binding pocket rather than a distant allosteric site; however, its anthraquinone scaffold appears to establish a binding interaction pattern distinct from endogenous estradiol. This non-classical engagement of the canonical pocket may underlie its ability to activate ESR1-linked hematopoietic signaling without behaving as a conventional steroidal estrogen. Compared with estradiol, the anthraquinone scaffold of emodin may confer a different conformational bias on ESR1, potentially favoring specific co-activator complexes or non-genomic signaling; this hypothesis warrants further investigation.

Given the pleiotropic pharmacology of emodin, ESR1 should not be interpreted as the only possible molecular target of this compound. In the present study, ESR1 was prioritized through network pharmacology and transcriptomic pathway analysis and then validated by docking, molecular dynamics simulation, CETSA/DARTS, pharmacological inhibition, and ESR1 siRNA knockdown. These data establish ESR1 as a functionally required target in emodin-induced MK differentiation. However, we did not perform a broad experimental selectivity screen against other nuclear receptors, kinases, or potential emodin-binding proteins. Therefore, other emodin-responsive targets may also contribute to its broader pharmacological actions, particularly in vivo. Future studies using nuclear receptor selectivity panels, kinome profiling, chemoproteomics, competitive binding assays, and systematic genetic loss-of-function approaches will be needed to define the full target spectrum of emodin and to distinguish ESR1-dependent from ESR1-independent mechanisms.

Downstream of ESR1, both our in silico analyses and experimental data point to PI3K/AKT and JAK2/STAT3 as key effector pathways. These cascades are well-established mediators of MK survival, endomitosis, and proplatelet formation and are classically activated by TPO/c-MPL [8, 28]. In our study, emodin enhanced phosphorylation of PI3K, AKT, JAK2, and STAT3, and increased expression and nuclear localization of NF-E2, EGR1, and RUNX1. Pharmacological blockade with LY294002 or ruxolitinib not only suppressed these signaling events but also abrogated the morphological and phenotypic hallmarks of emodin-induced MK maturation. These observations place PI3K/AKT and JAK2/STAT3 as indispensable components of the emodin–ESR1 signaling axis. The situation is conceptually similar to our previous work on bavachinin A, where FLT3 was identified as the primary binding target and PI3K/AKT as the core downstream pathway mediating MK differentiation and thrombopoiesis, independent of TPO/c-MPL [25]. Here, emodin recruits the same central signaling hubs, but through an entirely different membrane–nuclear receptor (ESR1) rather than a conventional hematopoietic tyrosine kinase receptor. The convergence of these distinct upstream receptors on PI3K/AKT and JAK2/STAT3 underscores that these pathways form a common “thrombopoietic core module” that can be engaged by multiple physiological and pharmacological inputs.

An important conceptual point emerging from this work is that emodin promotes megakaryopoiesis via an ESR1-centered, TPO/c-MPL–independent route. Our RIT data show clear efficacy even in the absence of exogenous TPO, and the literature cited above provides several precedents for non-TPO mechanisms—CCL5/CCR5, YRS^ACT^, and AVP-AKT–driven mitochondrial remodeling—that modulate MK biology under conditions of inflammation or stress [36–38]. By adding ESR1 to this network, our study broadens the spectrum of receptors that can be targeted to manipulate thrombopoiesis. Clinically, such alternative pathways are attractive because they may circumvent limitations of TPO-based therapies, such as refractoriness, risk of marrow fibrosis, and thrombosis [32–34]. At the same time, the estrogenic nature of ESR1 signaling raises obvious safety questions. Systemic ESR1 activation might influence reproductive tissues, breast or endometrial proliferation, and coagulation balance [11, 14, 39]. Whether emodin, as a non-steroidal ligand, displays a more favorable “selective estrogen receptor modulator–like” profile in hematopoietic tissues while sparing classical estrogen target organs is an important question for future work. Because ESR1 signaling is influenced by sex and hormonal status, this issue should be interpreted cautiously. In the present study, male KM mice were used to reduce variability associated with estrous-cycle-dependent estrogen fluctuations. However, this design does not address whether emodin exerts comparable thrombopoietic efficacy in female mice or whether estrous-cycle stage modifies ESR1-mediated hematopoietic responses. Future studies incorporating both sexes, estrous-cycle monitoring in female mice, and direct comparison of ESR1 signaling responses will be required to define potential sex-dependent effects of RR/emodin on thrombopoiesis. Isoform-specific effects (ERα vs ERβ), sex-dependent responses, hormonal status, and long-term safety during repeated administration therefore merit careful evaluation in future studies. The route of administration also represents a translational limitation. In this study, RR and emodin were delivered intraperitoneally to ensure stable systemic exposure during proof-of-concept efficacy and mechanistic evaluation. However, oral efficacy was not tested, and intraperitoneal injection is not intended to represent the final clinically preferred route. Given the traditional oral use of RR and the potential oral development of emodin, future studies should compare oral and intraperitoneal dosing, define pharmacokinetic/pharmacodynamic profiles, determine oral bioavailability, and optimize formulations suitable for clinical translation.

The apparent discrepancy between the in vitro screening results—where only emodin showed robust activity—and the in vivo efficacy of the whole RR extract also deserves comment. LC–MS/MS quantification showed that RR contained 0.44 mg/g emodin. Based on this value, the RR doses used in vivo corresponded to only 0.0088–0.0352 mg/kg emodin-equivalent exposure, which is far below the purified emodin doses required to produce robust thrombopoietic efficacy. This dose difference indicates that the in vivo activity of RR cannot be attributed solely to its emodin content. Rather, emodin should be viewed as a key MK-promoting constituent identified through functional screening, while other RR components may cooperate by modulating pharmacokinetics, metabolism, inflammatory responses, radiation-induced BM injury, or the hematopoietic microenvironment. Several RR components may be converted in vivo to metabolites with higher MK activity that our in vitro assays did not capture. In addition, multi-target actions, such as suppression of inflammatory cytokines, improvement of BM stromal support, or regulation of coagulation and vascular tone, may require combinations of compounds. Our network pharmacology analysis already hints at additional targets and pathways beyond ESR1 and PI3K/AKT–JAK2/STAT3. Future studies using fractionation, metabolite profiling, and combination index approaches will be needed to fully dissect the cooperative network within RR.

**Fig. 14:**
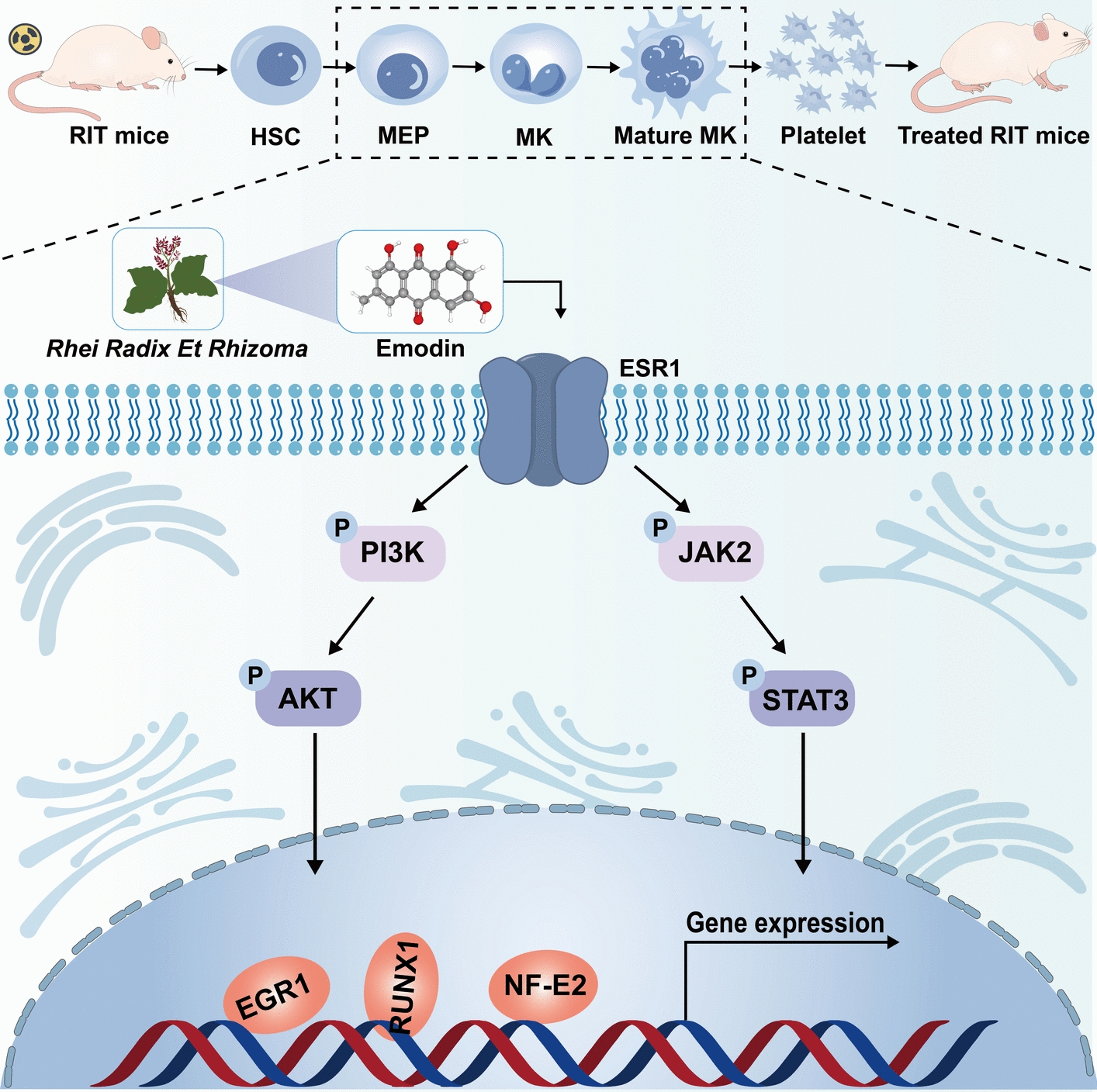
Schematic model of emodin-mediated ESR1 activation and downstream signaling in MK differentiation and thrombopoiesis. HSC: hematopoietic stem cell. MEP: megakaryocyte/erythroid progenitor

Taken together, the integrated picture that emerges from our data is coherent (Fig. 14). RR, a classical TCM herb, exerts potent anti-RIT effects through multi-component, multi-target actions. Emodin is the key megakaryopoiesis-promoting constituent that directly binds and activates ESR1 in MK-lineage cells. ESR1 activation engages PI3K/AKT and JAK2/STAT3 signaling, culminating in upregulation of the thrombopoietic transcription factors NF-E2, EGR1, and RUNX1, expansion of MK progenitors, enhanced polyploidization, and increased platelet production across BM, spleen, and lung. Other RR components may cooperate by protecting BM from radiation injury and modulating the hematopoietic microenvironment. Together, these mechanisms provide a rational basis for the observed improvement in platelet recovery and BM integrity in irradiated mice and position RR/emodin as promising candidates for further development as adjunctive therapies for RIT.

## Conclusion

In this study, we established a comprehensive research framework-from herbal extract to active monomer, from phenotypic validation to target discovery, and from computational prediction to multi-level mechanistic verification-to elucidate the therapeutic value and molecular basis of RR in the treatment of RIT. This systematic approach allowed us not only to define the hematopoietic benefits of RR but also to pinpoint emodin as its principal megakaryopoiesis-promoting constituent and to uncover a previously unrecognized regulatory axis governing thrombopoiesis. Through integrated in vitro* and *in vivo analyses, we revealed that emodin enhances platelet regeneration while mitigating radiation-induced BM injury, and that it restores megakaryopoiesis across multiple hematopoietic organs. Mechanistically, the study identifies ESR1 as a new, non-steroidal molecular target directly engaged by emodin, supported by docking, molecular dynamics, and CETSA/DARTS assays. Activation of ESR1 subsequently triggers the PI3K/AKT and JAK2/STAT3 pathways, driving progenitor expansion, megakaryocyte maturation, and polyploidization. Importantly, this mechanism operates independently of the classical TPO/c-MPL axis, highlighting an alternative signaling route that can be exploited for therapeutic intervention. Collectively, the study uncovers mechanism for the thrombopoietic effects of RR, establishes emodin as a promising lead compound for RIT therapy, and reveals an ESR1-centered signaling pathway as a novel regulatory mechanism of megakaryopoiesis. These findings offer new opportunities for the development of non-TPO–based thrombopoietic agents derived from natural products.

## Supplementary Information

Supplementary Material 1.

## Data Availability

All data supporting the findings of this study are available within the paper and its Supplementary Information.

## Abbreviations

TPO: Thrombopoietin
rhTPO: Recombinant human TPO
BM: Bone marrow
MK: Megakaryocyte
TCM: Traditional Chinese Medicine
RIT: Radiation-induced thrombocytopenia
RR: *Rhei Radix et Rhizoma*
UHPLC–HRMS: Ultra-high performance liquid chromatography–high-resolution mass spectrometry
IDA: Information-dependent acquisition
HSPCs: Haematopoietic stem and progenitor cells
CCK-8: Cell Counting Kit-8
LDH: Lactate dehydrogenase
BSA: Bovine serum albumin
KM: Kunming
WBC: White blood cells
RBC: Red blood cells
H&E: Hematoxylin and eosin
ALT: Alanine aminotransferase
AST: Aspartate aminotransferase
CK: Creatine kinase
TTD: Therapeutic Target Database
PPI: Protein–protein interaction
GO: Gene Ontology
KEGG: Kyoto Encyclopedia of Genes and Genomes
DEGs: Differentially expressed genes
RMSD: Root mean square deviation
RMSF: Root mean square fluctuation
CETSA: Cellular thermal shift assay
DARTS: Drug affinity responsive target stability
SD: Standard deviation
TIC: Total ion chromatogram
YRS^ACT^: TyrosyltRNA synthetase

## References

[1] Yu W, Jiao K, Wang K, Li X, Wan Q, Qin W, et al. Metabolic support protects mucosa from ferroptosis in radiation-induced mucositis. Nat Commun. 2025. 10.1038/s41467-025-67214-5. 41361241 10.1038/s41467-025-67214-5 PMC12804980

[2] Du C, Liu C, Yu K, Zhang S, Fu Z, Chen X, et al. Mitochondrial serine catabolism safeguards maintenance of the hematopoietic stem cell pool in homeostasis and injury. Cell Stem Cell. 2024;31:1484-1500.e9. 10.1016/j.stem.2024.07.009. 39181130 10.1016/j.stem.2024.07.009

[3] Takeda K, Umezawa R, Yamamoto T, Takahashi N, Suzuki Y, Kishida K, et al. Acute hematologic toxicity of radiation therapy – a comprehensive analysis and predictive nomogram. J Radiat Res. 2023;64:954–61. 10.1093/jrr/rrad069. 37740569 10.1093/jrr/rrad069 PMC10665302

[4] Mei J, Jiao F, Li Y, Cui J, Yang H, Wang L. Application of thrombopoietic agents in cancer therapy-induced thrombocytopenia: a comprehensive review. Blood Rev. 2025;70:101257. 10.1016/j.blre.2025.101257. 39809679 10.1016/j.blre.2025.101257

[5] Merli P, Strocchio L, Vinti L, Palumbo G, Locatelli F. Eltrombopag for treatment of thrombocytopenia-associated disorders. Expert Opin Pharmacother. 2015;16:2243–56. 10.1517/14656566.2015.1085512. 26364898 10.1517/14656566.2015.1085512

[6] Tarantino MD, Mosalpuria K, Kolodny S, Zhang J, Vredenburg M, Jamieson BD. Safety, efficacy, and treatment satisfaction in adults with ITP who switched to avatrombopag from another TPO-RA. Blood Adv. 2025;9:2733–43. 10.1182/bloodadvances.2024015635. 40101243 10.1182/bloodadvances.2024015635 PMC12166367

[7] Fu W, Ishikawa-Ankerhold H, Gaertner F. Homeostasis of megakaryocytes: balancing tissue residency and consumptive platelet production. Trends Cell Biol. 2025. 10.1016/j.tcb.2025.11.002. 41330800 10.1016/j.tcb.2025.11.002

[8] Tsutsumi N, Masoumi Z, James SC, Tucker JA, Winkelmann H, Grey W, et al. Structure of the thrombopoietin-MPL receptor complex is a blueprint for biasing hematopoiesis. Cell. 2023;186:4189-4203.e22. 10.1016/j.cell.2023.07.037. 37633268 10.1016/j.cell.2023.07.037 PMC10528194

[9] Chapple RH, Hu T, Tseng Y-J, Liu L, Kitano A, Luu V, et al. ERα promotes murine hematopoietic regeneration through the Ire1α-mediated unfolded protein response. Elife. 2018;7:e31159. 10.7554/eLife.31159. 29451493 10.7554/eLife.31159 PMC5829925

[10] Du C, Xu Y, Yang K, Chen S, Wang X, Wang S, et al. Estrogen promotes megakaryocyte polyploidization via estrogen receptor beta-mediated transcription of GATA1. Leukemia. 2017;31:945–56. 10.1038/leu.2016.285. 27748371 10.1038/leu.2016.285

[11] Gui Z, Shi W, Zhou F, Yan Y, Li Y, Xu Y. The role of estrogen receptors in intracellular estrogen signaling pathways, an overview. J Steroid Biochem Mol Biol. 2025;245:106632. 10.1016/j.jsbmb.2024.106632. 39551163 10.1016/j.jsbmb.2024.106632

[12] Liu R, Wang C, Huang D, Che H, Piao Y, Hong C, et al. Lurongdabu Decoction alleviates mitochondrial damage and preserves nasal mucosal barrier integrity via the ESR1/PI3K/AKT and EGFR/FAK/SRC signaling pathways. J Ethnopharmacol. 2026;357:120911. 10.1016/j.jep.2025.120911. 41270909 10.1016/j.jep.2025.120911

[13] Yang F, Lai J, Deng J, Du J, Du X, Zhang X, et al. The application of ethnomedicine in modulating megakaryocyte differentiation and platelet counts. Int J Mol Sci. 2023;24:3168. 10.3390/ijms24043168. 36834579 10.3390/ijms24043168 PMC9961075

[14] Chen W, Zhu L, Wang L, Zeng J, Wen M, Xu X, et al. A novel antithrombocytopenia agent, rhizoma cibotii, promotes megakaryopoiesis and thrombopoiesis through the PI3K/AKT, MEK/ERK, and JAK2/STAT3 signaling pathways. Int J Mol Sci. 2022;23:14060. 10.3390/ijms232214060. 36430539 10.3390/ijms232214060 PMC9694118

[15] Yang X, Wang L, Zeng J, Wu A, Qin M, Wen M, et al. Caulis polygoni multiflori accelerates megakaryopoiesis and thrombopoiesis via activating PI3K/Akt and MEK/ERK signaling Pathways. Pharmaceuticals (Basel). 2022;15:1204. 10.3390/ph15101204. 36297316 10.3390/ph15101204 PMC9607024

[16] Wang L, Liu S, Luo J, Mo Q, Ran M, Zhang T, et al. Targeting a thrombopoietin-independent strategy in the discovery of a novel inducer of megakaryocytopoiesis, DMAG, for the treatment of thrombocytopenia. haematol. 2022;108:1394–411. 10.3324/haematol.2022.282209. 10.3324/haematol.2022.282209 PMC10153531 36546424

[17] Jiang X, Sun Y, Yang S, Wu Y, Wang L, Zou W, et al. Novel chemical-structure TPOR agonist, TMEA, promotes megakaryocytes differentiation and thrombopoiesis via mTOR and ERK signalings. Phytomedicine. 2023;110:154637. 10.1016/j.phymed.2022.154637. 36610353 10.1016/j.phymed.2022.154637

[18] Qiu Q, Fu F, Wu Y, Han C, Pu W, Wen L, et al. Rhei Radix et rhizoma and its anthraquinone derivatives: Potential candidates for pancreatitis treatment. Phytomedicine. 2024;129:155708. 10.1016/j.phymed.2024.155708. 38733906 10.1016/j.phymed.2024.155708

[19] Xu H, Wang W, Li X, Li Y, Jiang Y, Deng C, et al. Botany, traditional use, phytochemistry, pharmacology and clinical applications of rhubarb (rhei radix et rhizome): a systematic review. Am J Chin Med. 2024;52:1925–67. 10.1142/S0192415X24500757. 39558546 10.1142/S0192415X24500757

[20] Chen P, Gong Q, Wang H, Wang C, Wang W, Wu J, et al. Analgesic mechanism of emodin in neuropathic pain through inhibiting P2X4 purinoceptor signaling. Mol Neurobiol. 2025;62:10210–27. 10.1007/s12035-025-04906-5. 40195215 10.1007/s12035-025-04906-5

[21] Dong X, Fu J, Yin X, Cao S, Li X, Lin L, et al. Emodin: A review of its pharmacology, toxicity and pharmacokinetics. Phytother Res. 2016;30:1207–18. 10.1002/ptr.5631. 27188216 10.1002/ptr.5631 PMC7168079

[22] Semwal RB, Semwal DK, Combrinck S, Viljoen A. Emodin - a natural anthraquinone derivative with diverse pharmacological activities. Phytochemistry. 2021;190:112854. 10.1016/j.phytochem.2021.112854. 34311280 10.1016/j.phytochem.2021.112854

[23] Shen Z, Zhao L, Yoo S, Lin Z, Zhang Y, Yang W, et al. Emodin induces ferroptosis in colorectal cancer through NCOA4-mediated ferritinophagy and NF-κb pathway inactivation. Apoptosis. 2024;29:1810–23. 10.1007/s10495-024-01973-2. 38704789 10.1007/s10495-024-01973-2

[24] Tang X, Liao R, Zhou L, Yi T, Ran M, Luo J, et al. Genistin: a novel estrogen analogue targeting ERβ to alleviate thrombocytopenia. Int J Biol Sci. 2024;20:2236–60. 10.7150/ijbs.90483. 38617546 10.7150/ijbs.90483 PMC11008259

[25] Wang L, Zhang T, Yang X, Mo Q, Ran M, Li R, et al. Multimodal discovery of bavachinin a: a natural FLT3 agonist for treating thrombocytopenia. Phytomedicine. 2025;140:156597. 10.1016/j.phymed.2025.156597. 40058315 10.1016/j.phymed.2025.156597

[26] Cenariu D, Iluta S, Zimta A-A, Petrushev B, Qian L, Dirzu N, et al. Extramedullary hematopoiesis of the liver and spleen. J Clin Med. 2021;10:5831. 10.3390/jcm10245831. 34945127 10.3390/jcm10245831 PMC8707658

[27] Klairmont MM, Hoskoppal D, Yadak N, Choi JK. The comparative sensitivity of immunohistochemical markers of megakaryocytic differentiation in acute megakaryoblastic leukemia. Am J Clin Pathol. 2018;150:461–7. 10.1093/ajcp/aqy074. 30052718 10.1093/ajcp/aqy074

[28] Noetzli LJ, French SL, Machlus KR. New insights into the differentiation of megakaryocytes from hematopoietic progenitors. Arterioscler Thromb Vasc Biol. 2019;39:1288–300. 10.1161/ATVBAHA.119.312129. 31043076 10.1161/ATVBAHA.119.312129 PMC6594866

[29] Yang J, Luan J, Shen Y, Chen B. Developments in the production of platelets from stem cells (review). Mol Med Rep. 2020;23:1–1. 10.3892/mmr.2020.11645. 33179095 10.3892/mmr.2020.11645 PMC7673345

[30] Lefrançais E, Ortiz-Muñoz G, Caudrillier A, Mallavia B, Liu F, Sayah DM, et al. The lung is a site of platelet biogenesis and a reservoir for haematopoietic progenitors. Nature. 2017;544:105–9. 10.1038/nature21706. 28329764 10.1038/nature21706 PMC5663284

[31] Soliman DS, Al-Sabbagh A, Ibrahim F, El-Omri H, Yassin MA, Amer A. Local field radiotherapy induced therapy related myeloid neoplasms and bone marrow suppression. Blood. 2019;134:5113–5113. 10.1182/blood-2019-131683.

[32] Ghanima W, Cooper N, Rodeghiero F, Godeau B, Bussel JB. Thrombopoietin receptor agonists: ten years later. Haematologica. 2019;104:1112–23. 10.3324/haematol.2018.212845. 31073079 10.3324/haematol.2018.212845 PMC6545830

[33] Li G, Huang A, Yang Q, Chen Y, Jiang Y, Liu Z. Effectiveness and safety of hetrombopag in the management of radiotherapy-induced thrombocytopenia in patients with gynecological malignancies. Blood. 2023;142:5431–5431. 10.1182/blood-2023-182634.

[34] Xing L, Shi H, Xu R, Ye H, Hu M. Real-world effectiveness of romiplostim N01 with or without TPO-RA for cancer therapy-induced thrombocytopenia in cancer patients. Blood. 2025;146:6585–6585. 10.1182/blood-2025-6585.

[35] Yadav M, Song F, Huang J, Chakravarti A, Jacob NK. *Ocimum* flavone orientin as a countermeasure for thrombocytopenia. Sci Rep. 2018;8:5075. 10.1038/s41598-018-23419-x. 29567949 10.1038/s41598-018-23419-x PMC5864743

[36] Chen S, Sun K, Xu B, Han S, Wang S, Xu Y, et al. Akt-mediated mitochondrial metabolism regulates proplatelet formation and platelet shedding post vasopressin exposure. J Thromb Haemost. 2023;21:344–58. 10.1016/j.jtha.2022.11.018. 36700501 10.1016/j.jtha.2022.11.018

[37] Kanaji T, Vo M-N, Kanaji S, Zarpellon A, Shapiro R, Morodomi Y, et al. Tyrosyl-tRNA synthetase stimulates thrombopoietin-independent hematopoiesis accelerating recovery from thrombocytopenia. Proc Natl Acad Sci USA. 2018. 10.1073/pnas.1807000115. 30104364 10.1073/pnas.1807000115 PMC6126720

[38] Machlus KR, Johnson KE, Kulenthirarajan R, Forward JA, Tippy MD, Soussou TS, et al. CCL5 derived from platelets increases megakaryocyte proplatelet formation. Blood. 2016;127:921–6. 10.1182/blood-2015-05-644583. 26647394 10.1182/blood-2015-05-644583 PMC4760093

[39] Grinshpun A, Chen V, Sandusky ZM, Fanning SW, Jeselsohn R. ESR1 activating mutations: from structure to clinical application. Biochimica et Biophysica Acta (BBA)-Rev Cancer. 2023;1878:188830. 10.1016/j.bbcan.2022.188830. 10.1016/j.bbcan.2022.188830 PMC13423459 36336145

