# Supplementary material for: A systems herbal-to-molecule and transcriptomic strategy identifies emodin as an ESR1-targeting phytochemical driving thrombopoiesis
Source: Chin Med. 2026 Jul 6;21:180. doi: 10.1186/s13020-026-01454-5 (PMC13335142; doi:10.1186/s13020-026-01454-5)
Supplement: Supplementary file 1 — Supplementary Material 1. [file 13020_2026_1454_MOESM1_ESM.docx]

**A systems herbal-to-molecule and multi-omics approach identifies emodin as a novel ESR1-targeting phytochemical driving thrombopoiesis**

Xiao Qi ^a,1^, Qinyao Li ^b,1^, Fengyu Li ^a,1^, Linglin Zhou ^a^, Qi Mo ^c^, Jing Zeng ^a^, Tianci Hu ^a^, Sheng Liu ^a^, Xinyue Mei ^a^, Min Wu ^a^, Xuejing Qiang ^a^, Qiyang Cheng ^a^, Anguo Wu ^a^, Xiaogang Zhou ^a^, Feihong Huang ^a^, Qiaozhi Wang ^e^, Peng Chen ^d*^, Jianming Wu ^e,a,f, **^, and Long Wang ^a,***^

This file includes:

Supplementary Figures





**Supplementary Fig. 1.** Quantification of emodin in RR by LC-MS/MS. (A) Multiple reaction monitoring (MRM) chromatogram of RR. (B) MRM chromatogram of standard emodin solution (2 μg/mL) detected under the same LC-MS/MS conditions.
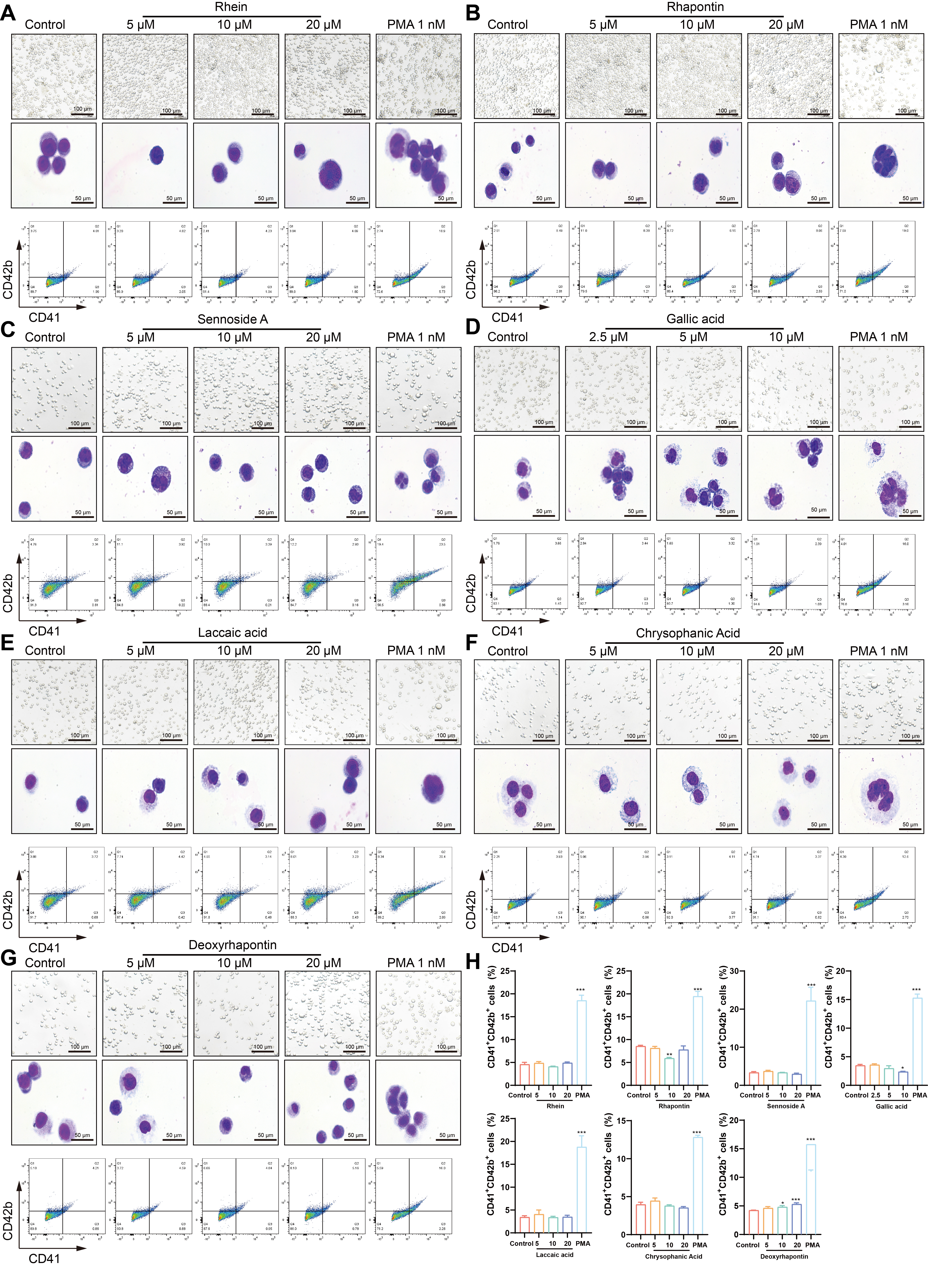


**Supplementary Fig. 2.** Screening of RR-derived phytochemicals for MK differentiation activity in Meg-01 cells. (A-G) Meg-01 cells were treated with increasing concentrations (5, 10, and 20 μM) of Rhein(A), rhapontin (B), sennoside A (C), gallic acid (D), laccaic acid (E), chrysophanic acid (F), and deoxyrhapontin (G), with PMA (1 nM) serving as a positive control. For each compound, phase-contrast microscopy, Giemsa staining, and flow cytometric profiling of CD41 and CD42b expression were performed to assess morphological and immunophenotypic features of MK differentiation. (H) Quantitative summary of CD41⁺CD42b⁺ cell percentages across all tested compound (n = 3). Data are presented as mean ± SD. Statistical signiﬁcance was determined using one-way ANOVA. **p* < 0.05, ***p* < 0.01, ****p* < 0.001 vs. control group.





**Supplementary Fig. 3.** Cytotoxicity and apoptosis assessment of emodin in Meg-01 and K562 cells. (A) CCK-8 assay of Meg-01 cells treated with emodin (2.5, 5, and 10 μM) for 1, 3, and 5 days, showing relative cell proliferation (% of control) (n = 3). (B) CCK-8 assay of K562 cells under the same treatment conditions as in (A) (n = 3). (C) LDH release in Meg-01 cells treated with emodin (2.5, 5, and 10 μM) for 1, 3, and 5 days; maximum LDH release was used as a positive control (n = 3). (D) LDH release in K562 cells under the same treatment conditions as in (C) (n = 3). (E) Annexin V/PI flow cytometric analysis of apoptosis in Meg-01 cells treated with emodin (2.5, 5, and 10 μM), with corresponding quantification of apoptotic rates (n = 3). (F) Annexin V/PI flow cytometric analysis of apoptosis in K562 cells treated as in (E), with quantification of apoptotic rates (n = 3). Data are presented as mean ± SD. For A-D, statistical signiﬁcance was determined using two -way ANOVA; for E-F, one -way ANOVA was used. **p* < 0.05, ***p* < 0.01, ****p* < 0.001 vs. control group.


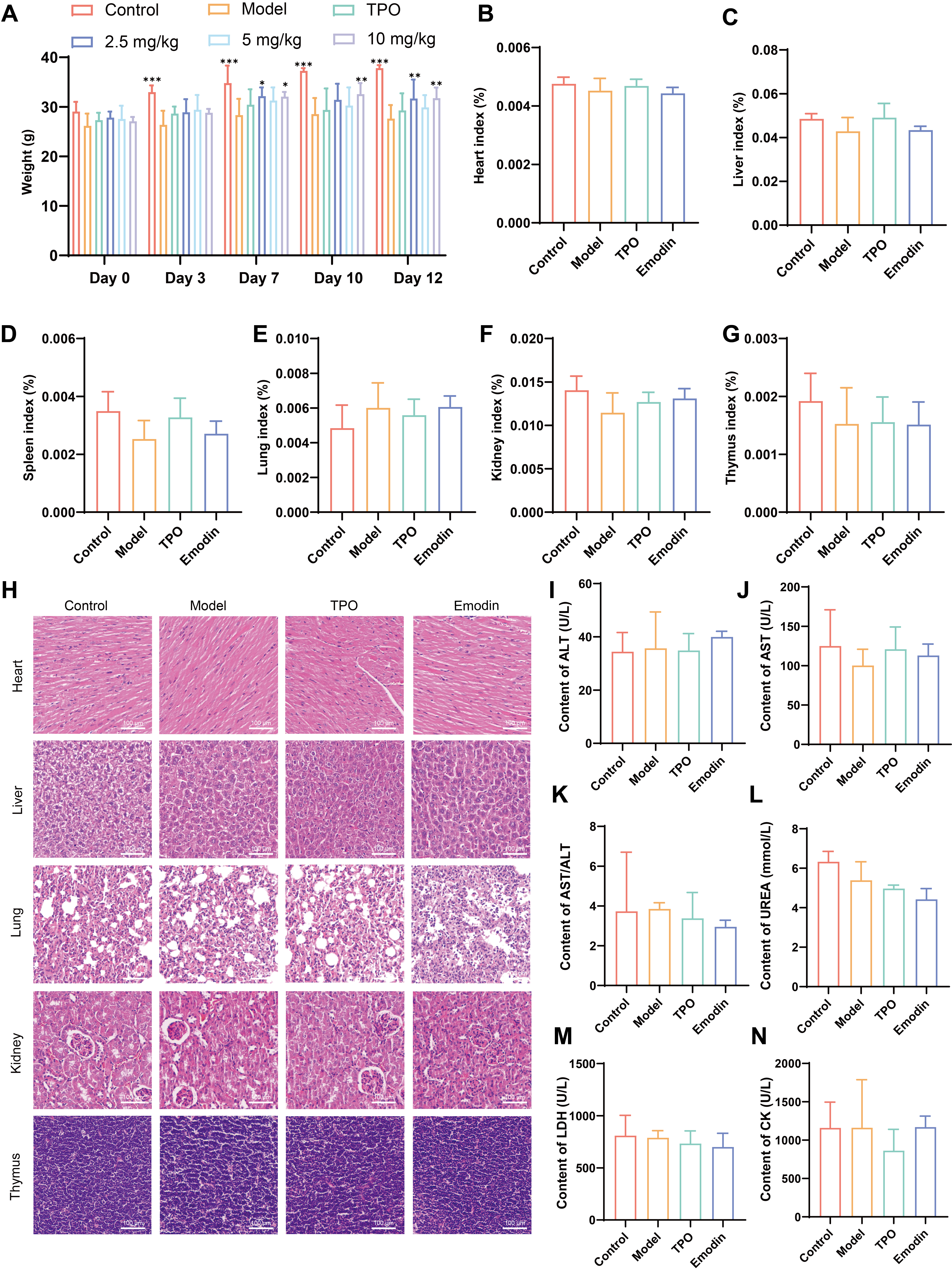


**Supplementary Fig. 4.** *In vivo* safety evaluation of emodin in irradiated mice. (A) Changes in body weight of mice following irradiation and subsequent treatment (n = 8). (B–G) Relative organ indices of the heart (B), liver (C), spleen (D), lung (E), kidney (F), and thymus (G) (n = 3). (H) Representative H&E staining of heart, liver, lung, kidney, and thymus tissues from each group. (I–N) Serum biochemical parameters, including ALT (I), AST (J), AST/ALT ratio (K), UREA (L), LDH (M), and CK (N) (n = 3). Data are presented as mean ± SD. For B-G, I-N statistical significance was determined using one-way ANOVA; for A, two-way ANOVA was used. **p* < 0.05, ***p* < 0.01, ****p* < 0.001 vs. model group.


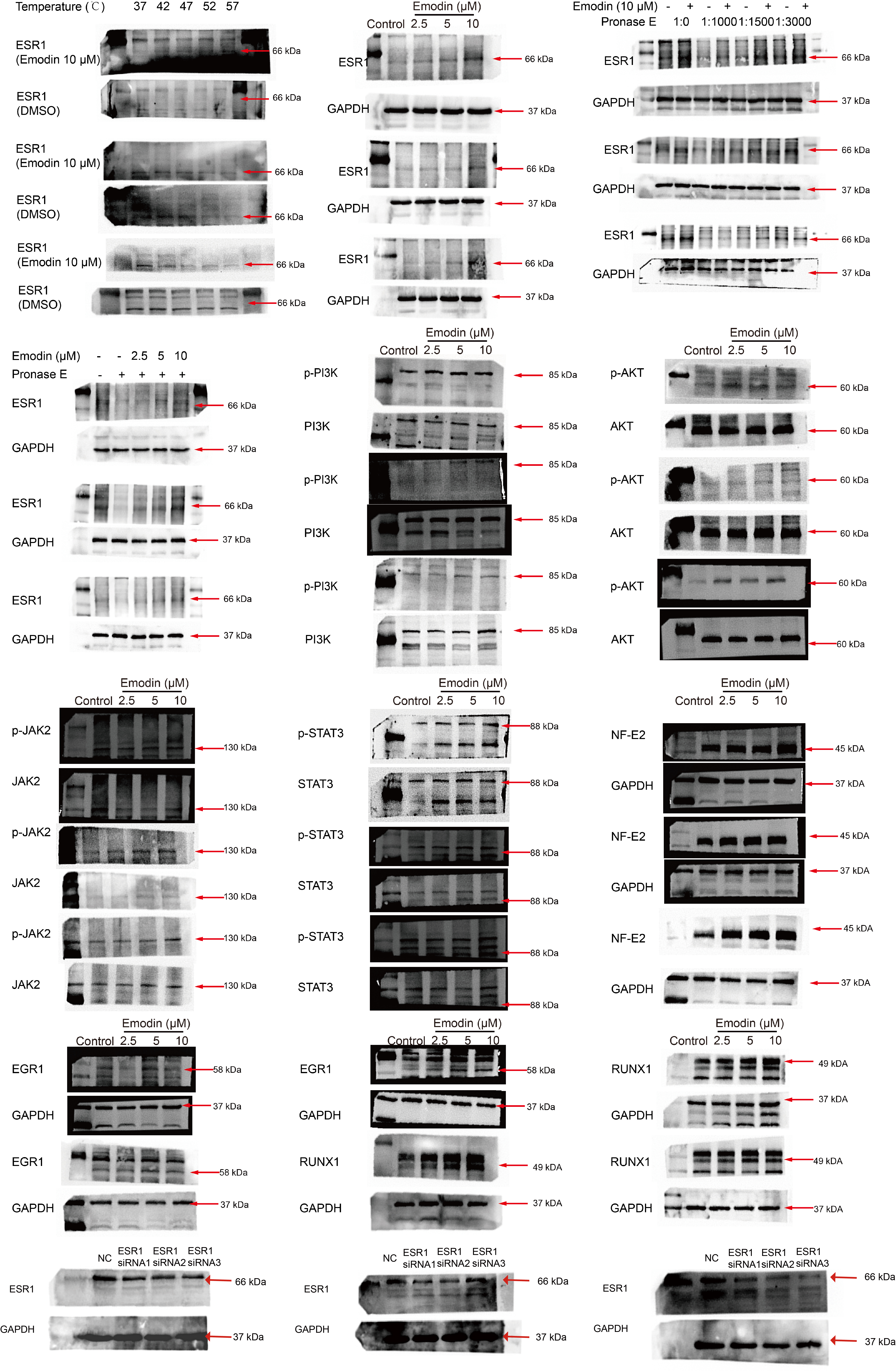
**Supplementary Fig. 5.** The source data for the western blot analysis presented in Figures 10-12.


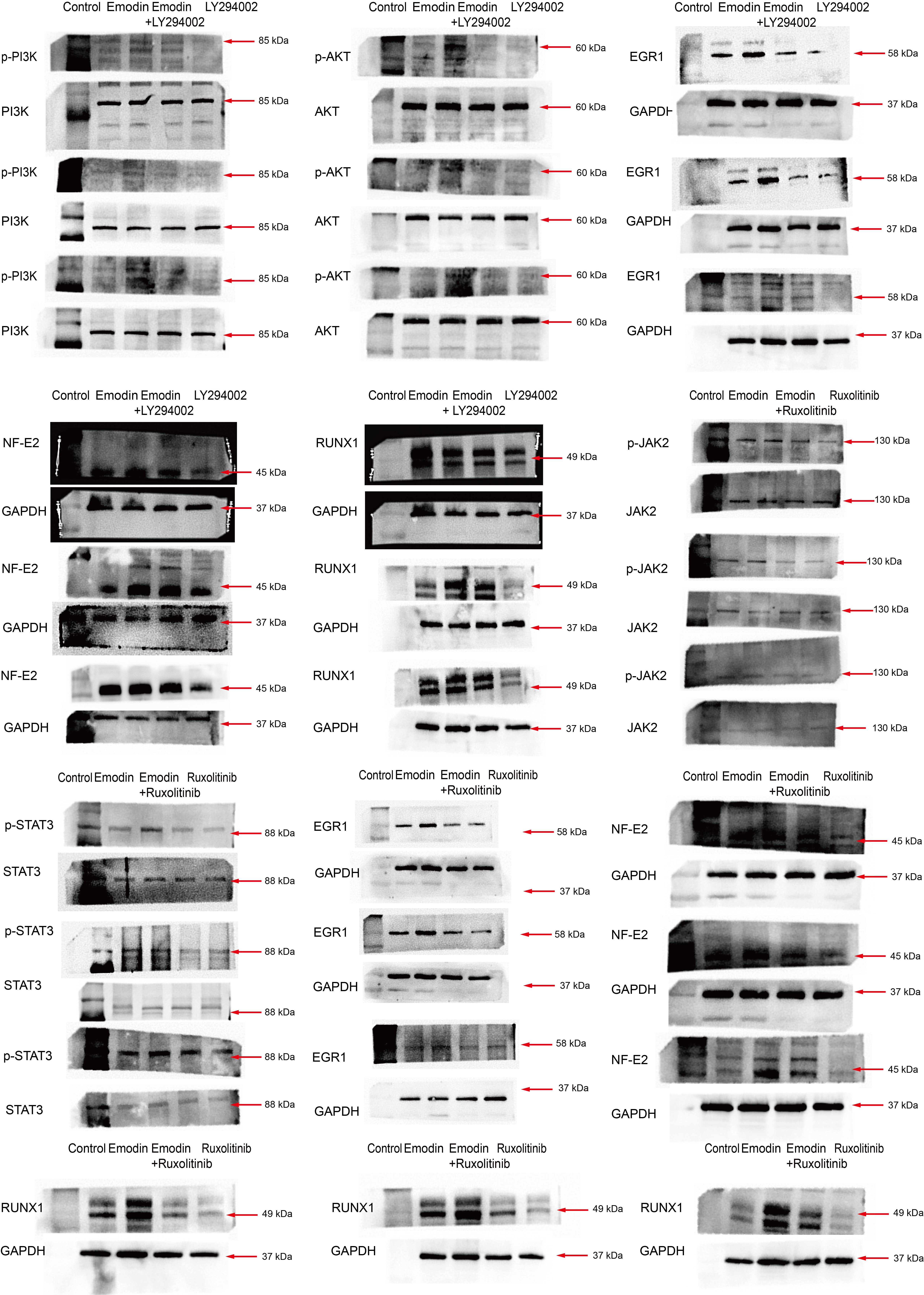


**Supplementary Fig. 6.** The source data for the western blot analysis presented in Figure 13.



**Supplementary Fig. 7.** The source data for the western blot analysis presented in Figure 13.
